# Astrocyte-neuron combined targeting for CYP46A1 gene therapy in Huntington’s disease

**DOI:** 10.1186/s40478-025-02054-4

**Published:** 2025-08-26

**Authors:** Louis-Habib Parsai, Farah Chali, Enejda Subashi, Caroline Zeitouny, Emilie Rey, A. Berniard, William Bitton, Laureline Urli, Lisa Rousselot, Nadège Sarrazin, Véronique Blouin, Wilfried F. A. Den Dunnen, Kristin Michaelsen-Preusse, Martin Korte, Sandro Alves, Nathalie Cartier

**Affiliations:** 1 NeuroGenCell, Inserm U 1127, CNRS UMR 7225, ICM, Institut du Cerveau, Sorbonne Université, Paris, France; 2 AskBio, Inc., Research Triangle Park, 20 TW Alexander Dr, NC 27703 Durham, North Carolina, USA; 3 Department of Cellular Neurobiology, Zoological Institute, Technische Universität Braunschweig, 38106 Brunswick, Germany; 4 Plateforme PHENO-ICMice, Inserm U1127, CNRS UMR 7225, ICM, Institut du Cerveau (ICM), 75013 Paris, France; 5 INSERM UMR 1089-CPV IRS 2 Nantes Biotech - Université de Nantes, Nantes, France; 6 Department of Pathology and Medical Biology, University Medical Centre Groningen, University of Groningen, 9700 RB Groningen, The Netherlands; 7 Neuroinflammation and Neurodegeneration, Helmhotlz Center for Infection Research, 38124 Brunswick, Germany

**Keywords:** Cholesterol, Astrocytes, Neurons, Synapses, Inflammation

## Abstract

Huntington’s disease (HD) is an autosomal dominant neurodegenerative disease caused by an abnormal expansion of cytosine-adenine-guanosine (CAG) trinucleotidein the huntingtin gene. Mutant huntingtin (mHTT) expression in neurons and glial cells affects neuron and astrocyte functions and leads to the loss of medium spiny neurons of the striatum. Brain cholesterol pathway is severely affected by HTT mutation in neurons and astrocytes, contributing to HD pathogenesis. Decreased cholesterol production and transport by astrocytes impair synapse maturation and neurotransmission. Brain cholesterol metabolism is maintained by cholesterol hydroxylation into 24-hydroxycholesterol by the neuronal enzyme cholesterol 24-hydroxylase (CYP46A1). CYP46A1 is decreased in affected brain regions in HD patients and mice. AAV-CYP46A1 striatal delivery was shown to restore cholesterol metabolism with neuroprotective effects in two mouse models of HD, characterized by mHTT aggregates’ reduction, improved transcriptomic profile, and Brain-Derived Neurotrophic Factor (BDNF) signaling, and preservation of striatal neurons. From a therapeutic perspective, we intended to clarify the detailed mechanisms and the specific role of neurons and astrocytes in the therapeutic effects of AAV-CYP46A1 delivery. We first evaluated CYP46A1 expression in astrocytes in HD post-mortem putamen at a late stage of disease progression. To determine the specific contribution of CYP46A1 expression in astrocytes compared to neurons on the HD phenotype, we assessed the effects of AAV-CYP46A1 striatal injection under the control of astrocytic (GFA2) or neuronal (hSYN) promoters in R6/2 mice. Overall, equivalent transgenic CYP46A1 protein levels, both astrocytic and neuronal targeting, mitigate medium ppiny neuron (MSN) atrophy and improve spine density in R6/2 mice. Reduction of mHTT aggregates in neurons is similar when CYP46A1 is overexpressed in neurons or in astrocytes. However, astrocyte targeting reduces mHTT aggregates in neurons and astrocytes, while restricted neuronal targeting reduces mHTT aggregates in neurons only. Altogether, astrocytic targeting of CYP46A1 expression in CYP46A1-tested animals combines cell-autonomous and non-cell-autonomous mechanisms of action, with improved phenotypic correction compared to neuronal-restricted targeting. Allowing expression in both cell types with higher expression levels of CYP46A1 showed overall better efficacy. We demonstrate that astrocyte-neuron combined targeting with AAV-CAG-CYP46A1 delivery increases therapeutic efficacy. This study brings new evidence that CAG-mediated CYP46A1 striatal overexpression significantly modifies the transcriptome in R6/2 mice for pathways involved in synaptogenesis and inflammation, suggesting targeting both astrocytes and neurons provides benefits for HD phenotypic correction.

**Supplementary Information:**

The online version contains supplementary material available at 10.1186/s40478-025-02054-4.

## Introduction

Huntington’s disease (HD) is a progressive, fatal and dominantly inherited neurodegenerative disease, characterized by a triad of cognitive, motor and psychiatric symptoms for which no curative therapy is available [1, 2]. HD is caused by an atypical CAG repeat expansion in the huntingtin gene (*HTT*) coding for a polyglutamine expansion in the mutant HTT protein. (mHTT) [3] This mutant protein manifests both a toxic gain of function and loss of function in normal HTT [4], exerting its impact ubiquitously throughout the body. The striatum is highly vulnerable, leading to progressive and severe atrophy [5, 6].

While the accumulation of mHTT in neurons [7] is acknowledged as a primary contributor to the privileged loss of striatal medium spiny-neurons (MSNs) [5, 6], growing evidence underscore the involvement of defective astrocytes in striatal neuron dysfunction and subsequent loss in HD. Intriguingly, mHTT is not confined to neurons but also extends its expression to glial cells in HD patients [8] and in HD mouse models [8–11],emphasizing the intricate role of glia, particularly astrocytes in HD pathogenesis.

Expression of mHTT specifically in astrocytes leads to an HD-like phenotype in an HD mouse model [8, 9] while its deletion slowed the progression of some disease-related symptoms [12]. Transplanted normal glia can improve disease phenotype in transgenic HD mice and mHTT glia can impact the disease phenotype of normal mice, thus suggesting a causal role for glia in HD [13]. Interestingly, while lowering HTT only in neurons was insufficient to rescue the phenotype of YAC 128 mice, lowering HTT in both neurons and astrocytes allowed for optimal functional benefit [14]. Indeed, astrocytes play a key role in supporting neuronal functions such as synaptic transmission and plasticity, metabolism, trophic support, antioxidant shielding and, particularly, cholesterol metabolism [15].

Brain cholesterol metabolism is required for optimal synaptogenesis, synaptic activity and central nervous system (CNS) development [16, 17].

In the adult brain, cholesterol is mostly synthesized in situ by astrocytes and transported to neurons. Mutation of genes implicated in cholesterol synthesis or transport leads to abnormal brain development, impaired cognitive functions, and neurodegeneration [18, 19].

Disrupted brain cholesterol homeostasis plays a detrimental role in HD, with decreased brain sterol synthesis and accumulation of cholesterol in neuronal membranes [20, 21]. mRNA levels of key enzymes involved in cholesterol synthesis are reduced in mHTT inducible cells lines, HD mouse models and post-mortem brain from HD patients [22–25]. In mHTT-expressing astrocytes in HD mice, mHTT interacts with and sequesters sterol regulatory element-binding protein-2 (SREBP-2)/importin-β complex in the cytoplasm hindering its maturation and nuclear translocation [24–26]. Consequently, mHTT diminishes the expression of downstream targets including the rate limiting enzyme the 3-hydroxy-3-methylglutaryl-coenzyme A reductase (HMGCR), in cholesterol biosynthesis [27]. Notably, a recent study reported that specific overexpression of SREBP2 in astrocytes in R6/2 mice enhanced cholesterol biosynthesis pathway genes, cleared mHTT aggregates, rescued synaptic transmission and improved behavior [28]. Additionally, in various HD mouse models mHTT-expressing astrocytes exhibited reduced levels of ApoE and ABCA1 expression. This impairs the production and secretion of ApoE-lipoprotein-bound cholesterol leading to reduced cholesterol efflux to neurons [23, 25]. The landscape of cholesterol levels in HD brains is marked by conflicting findings. Indeed, several studies have reported decreased total cholesterol levels, in particular in the striatum, as observed through mass spectrometry [23, 28–33] while others demonstrate cholesterol accumulation in HD neurons *in vitro* [34, 35] and increased total cholesterol levels *in vivo* [36] or no observed changes [37]. Despite this, these data point to alterations in cholesterol biosynthesis in the brain that are linked to the neuronal dysfunction that characterizes HD.

Cholesterol cannot cross the blood–brain-barrier (BBB) and brain cholesterol is produced in situ. To maintain brain cholesterol homeostasis, cholesterol in excess is converted into 24-Refhydroxycholesterol (24-OHC) by the neuronal cholesterol 24-hydroxylase (CYP46A1) enzyme [38]. The major pathway for cholesterol catabolism is achieved by CYP46A1. CYP46A1 deficiency is reported in the brains of HD patients and mice [36, 37, 39] In addition, the cholesterol catabolite 24-OHC is decreased in the brain and the plasma from HD mice, post-mortem tissue and plasma from HD patients [23, 29, 31, 36, 37, 40, 41].

Additionally, decreased plasma levels of 24-OHC in HD patients are directly correlated with caudate atrophy [40, 41].

We previously showed that overexpression of CYP46A1 by intracerebral delivery of an AAV vector using CMV/β-actin hybrid promoter (CAG) promotes beneficial and neuroprotective effects in the R6/2 and zQ175 mouse models of HD [36, 37]. Considering the pivotal role of astrocytes in the physiopathology of HD, and in brain cholesterol metabolism, we hypothesized that the contribution of cholesterol metabolism restoration in astrocytes and the neuron/astrocyte interplay could be advantageous for further therapeutic intervention. The goal of this study was to elucidate the contribution of astrocytes and neurons with CYP46A1 expression, and, in particular, the astrocytic correction of HD. Towards this hypothesis we show that CYP46A1 is re-expressed in astrocyte-like cells, while its expression is decreased in neurons from HD patients striata [36], suggesting a compensatory mechanism.

To further decipher the specific role of cholesterol metabolism in astrocytes, we used an AAV vector to evaluate the consequence of CYP46A1 targeting in R6/2 mice specifically in astrocytes or in neurons. Our findings unveil that overexpressing CYP46A1 exclusively in astrocytes using the GFA2 promoter brings therapeutic benefits in HD mice. Notably, a synergistic improvement is achieved when CYP46A1 targeting extends to both neurons and astrocytes, facilitated by the use of the CAG promoter. This underscores the therapeutic relevance of a dual-targeted approach targeting simultaneously different cell populations.

Importantly, we demonstrate that specific CYP46A1 expression in astrocytes leads to decreased mHTT aggregates in both astrocytes and neurons, improves motor function, mitigates MSN atrophy, improves MSN spine density, and activates the cholesterol pathway with increased production of cholesterol precursors. Through transcriptomic studies we also characterized the role of CYP46A1 on inflammation, as highlighted by increased astrocyte reactivity associated. Moreover, we also emphasized the role of CYP46A1 on synaptogenesis as its effects correlate with consequent mitigation of MSN atrophy and rescue of spine density.

When restricting CYP46A1 overexpression to neurons using the synapsin promoter, a non-statistical increase for improved motor phenotype with significant mitigation of MSN atrophy and increased MSN spine density was observed. However, reduction of mHTT aggregates was only observed in neurons.

Altogether, these data demonstrate that combined expression of CYP46A1 in astrocytes and neurons is beneficial for optimal correction of the R6/2 severe phenotype. This study paves the way for a deeper understanding of the astrocytic contribution in HD pathogenesis and opens avenues for targeted therapeutic strategies.

## Materials and methods

### Postmortem brain samples from HD patients and control

#### Individuals

In this study, postmortem brains from five subjects with clinically, morphologically, and genetically diagnosed HD (carrying between 45 and 66 CAG repeats on the mutant allele; 5 females, age ranging at death from 25 to 71 years), along with the brains from three age-matched control individuals (2 females, 1 male, age at death ranging from 33 to 65 years) with no evidence of neurological disease (Table 1). The HD brains were donated after informed consent by the patients themselves during life and these donations were supported by the spouse and doctors of these patients. The Medical Ethical committee of the University Medical Centre Groningen, the Netherlands approved the brain donation procedure, as well as the donation form and patient information. Genetic diagnosis was carried out in all HD subjects by genotyping the DNA extracted from peripheral lymphocytes.

**Table 1:** Information of human samples

ID Human	Sex	Age at death	Vonsattel stage	CAG repeat on the pathologic allele
Control				
S18-10,029	Male	33 years	–	–
S18-10,033	Female	47 years	–	–
S18-10,038	Female	65 years	–	–
HD patients				
S17-10,083	Female	25 years	4	66
S14-10,052	Female	50 years	4	50
S16-10,017	Male	40 years	4	55
S16-10,051	Female	47 years	3	45
S15-10,028	Female	36 years	3	53

### Histology in postmortem brain samples from HD patients and controls

#### Tissue preparation

The brains of HD subjects and age-matched control individuals were fixed in a 4% phosphate-buffered, aqueous formaldehyde solution (pH 7.4). Thereafter, tissue blocks from the cerebral cortex and caudate-putamen of HD subjects’ brain (n = 5) and age-matched control individuals (n = 3), were embedded in paraffin to cut into 5 μm thick horizontal sections. HD patients with Stage 3 and Stage 4 from Vonsattel classification were used in this experiment [42]. Sections were transferred onto glass microscope slides (TOMO® adhesion microscope slides). The paraffin-embedded slices tissues were conserved at − 20 °C, until use. The slides containing paraffin-embedded caudo-putamaen tissue were deparaffinized and rehydrated by immersing them through the following solutions: xylene (three immersions of 5 min each), ethanol 100% (two immersions of 5 min each), ethanol 95% (two immersion of 5 min each), ethanol 70% (5 min immersion), ethanol 50% (5 min immersion), ethanol 30% and deionized water, for 5 min. Paraffin-embedded slices tissues were permeabilized with PBS containing 0.2% Triton™ X-100, for 45 min.

#### Immunohistochemistry in paraffin-embedded sections

The immunohistochemical procedure (described in [43]) was initiated by permeabilization with Phosphate-Buffered Saline (PBS) containing 0.2% Triton™ X-100 for 45 min. Then, antigens were retrieved by boiling the sections in 1X citrate buffer pH 6.0, in a microwave oven at 350 W followed by quenching endogenous peroxidase by incubating paraffin-embedded sections of caudo-putamen in hydrogen peroxide for 30 min at room-temperature (RT). Sections were then permeabilized and non-specific epitopes were blocked by incubation for 2 h at room-temperature in PBS containing 4% bovine serum albumin (BSA), 4% normal goat serum (NGS) and 0.1% Triton™ X-100. Sections were incubated for 48 h at 4 °C with the respective primary antibody (Table 2), washed three times, and then incubated with the appropriate biotinylated secondary antibodies. (Biotinylated goat anti-mouse or goat anti-rabbit antibodies; 1:500; Vector Laboratories Inc., West Grove, CA, USA) for 2 h, at RT. After three washes, bound antibodies were visualized by the ABC amplification system (Vectastain ABC kit, Vector Laboratories) and DAB tetrahydrochloride (peroxidase substrate kit, DAB, Vector Laboratories) as the substrate.

**Table 2:** Primary antibodies used in this study

Primary antibodies	Source	WB^b^	IHC/IF^a^
Rat anti-HA	Sigma-Aldrich	–	1/400
Rabbit anti-GFAP	Dako	–	1/3000
Chicken anti-GFAP	Dako	–	1/2000
Rabbit anti-NeuN	Abcam	–	1/2000
Rabbit anti-Olig2	Abcam	–	1/2000
Rabbit anti-Iba1	Dako	–	1/2000
Rabbit anti-S100b	Abcam	–	1/700
Rabbit anti-CYP46A1	Abcam	–	1/2000
Mouse anti-CYP46A1	Millipore	1/2000	–
Mouse anti-huntingtin protein, clone mEM48	Millipore	1/2000	–
Rabbit anti-vinculin	abcam	1/300	–

^a^IHC/IF: Immunohistochemistry/Immunofluorescence

^b^WB: Western Blot

#### Quantification of CYP46A1 immunoreactivity in neurons from HD patients and healthy controls

To evaluate the cytoplasmic immunoreactivity of CYP46A1 in HD patients versus age-matched controls, CYP46A1 immunostained sections were acquired on a slide scanner, (Axio Scan Z.1, Zeiss, Germany) equipped with a Colibri illumination system (Colibri 2, Zeiss) and an Orca Flash 4.0 Hamamatsu. A Plan-Apochromat 10 × /0.45 objective for pre-focusing and a Plan-Apochromat 20 × /0.8 objective for fine focus image acquisition were used. Further analysis was performed using the *ImageJ* version 1.53c (NIH, Bethesda, USA) IHC Toolbox plug-in. A region of interest (ROI) was selected in the striatum, where at least 50 cells could be counted. In each of these ROIs, Color Detection button was used from the IHC Tool Box plug-in, enabling filtering of the H&E counterstaining. For each image, regions of interest were drawn around the cytoplasm of the cells as well as the background. The images were transformed into 8-bit images to apply a threshold (default mode). A mean Integrated density (IntDen1) value was calculated for 50–85 cells per image as well as the mean Integrated density (IntDen2) of the background was subtracted from this mean for normalization to obtain the final mean value.

## Animals

All experiments were conducted in accordance with ethical standards, French and European regulations (Directive 2010/63/EU).

Experiments realized on female C57BL/6J mice were validated by the local ethical committee of MIRCen animal facilities (Fontenay-aux-Roses, France) under specific pathogen-free conditions. All animal procedures were approved by the local ethical committee, CEtEA DSV n°44 and the French Ministry of National Education, Higher Education and Research (reference number 17081–2018071911254524 v2). Mice were housed in a temperature-controlled and maintained on a 12 h light/dark cycle. Food and water were available ad libitum*.* Mice were grouped with a maximum of 5 animals per cage. In addition, 25 C57BL/6J female mice were used for tropism experiments and assessment of long-term effects of CYP46A1 expression.

Regarding experiments realized on male and female R6/2 mice and WT-littermates, all animal procedures and experiments were approved by the local ethical committee (Ethical Committee of IBPS, n° 05) and the French Ministry of National Education, Higher Education and Research (reference number APAFIS #12,412–2,017,113,010,191,653 v7) and were performed in accordance with the Guide for the Care and Use of Laboratory Animals (US National Institutes of Health). Mice were housed in a temperature-controlled and maintained on a 12 h light/dark cycle. Food and water were available ad libitum*.* Mice were regrouped with a maximum of 6 animals per cage.

## Generation of R6/2 mice

R6/2 [B6CBA-Tg (HDexon1) 62Gpb/1 J] mice, which express exon 1 of the human mutant Huntington’s disease gene containing 160 CAG repeats, under the control of the human IT15 promoter, and wild-type (WT) littermates were generated by crossing ovarian-transplanted hemizygous females with B6CBAFI/J males. The described breeding couples were obtained from Jackson Laboratories (Bar Harbor, ME, USA).

## Genotyping and selection of mice

All mice used in the study were from the first offspring and the genotype was verified by polymerase chain reaction (PCR) using genomic DNA extracted from tail. The number of CAG repeat length varies very little in the progeny from the first generation (http://chdifoundation.org/wp-content/uploads/HD_Field_Guide_040414.pdf) and can therefore be considered around 160 ± 5 repeat expansions for all the mice that were used in the study.

Four-week-old R6/2 mice (n = 89) and age-matched wild-type littermates (n = 16) were used in this study. We used males and females, as both sexes are affected in HD. Nevertheless, in humans, women present a more important neurological damage (in terms of motor performance and functionally than men highlight by a lower score (Unified Huntington’s Disease Rating Scale (UHDRS) as well as a more rapid progression of the disease [44, 45].

### Production of recombinant adeno-associated viral vectors

All AAV vectors were produced and purified by Atlantic Gene therapies (INSERM U1089, Nantes, France). The viral constructs for AAVrh10-CAG-HA-CYP46A1, AAVrh10-hSYN-HA-CYP46A1, and AAVrh10-GFA2-HA-CYP46A1 contained the expression cassette consisting of either the human *CYP46A1* cDNA followed by human influenza HemAgglutinin tag (HA), driven by either a CMV/β-actin hybrid promoter (CAG), human synapsin (hSYN) and the Glial Fibrillary Acidic 2 (GFA2) promoter surrounded by inverted terminal repeats of AAV2. The viral constructs for rhAAV2/10-CAG-non-coding-HA-hCYP46A1 contained a deletion of 145 bp in the expression cassette human CYP46A1, driven by CMV/β-actin hybrid promoter (CAG). This deletion occurs 7 amino acids after the HA tag, resulting in a codon stop, preventing the expression of hCYP46A1 protein from *hCYP46A1* mRNA. This batch will be noted CAG-non-coding or non-coding group.

### Stereotaxic injections of AAVs were performed in 3-month-old female C57BL/6J wild-type mice and 4-week-old male and female R6/2 mice

Mice were anesthetized by intraperitoneal injection of ketamine/xylazine (0.1/0.05 g/kg body weight) and positioned on a stereotaxic frame (KOPF, USA) equipped with a Hamilton syringe (1701 RN 10μL, Dutscher, USA) and a 32G needle (Dutscher, France). Recombinant vectors were diluted in dPBS Ca^2+^/Mg^2+^ and bilaterally injected into the mouse striatum. Two microliters of viral preparation (corresponding to 3.10^9^ vg/striatum) were injected into the left and right striatum at a rate of 0.2 µL/minute. The stereotaxic coordinates for injection sites were: 1 mm rostral to the bregma, 2 mm lateral to midline and 3.5 mm ventral to the skull surface. The rate of injection was 0.2 μL/min with a total volume of 2 μL per striatum. In addition, wild-type C57BL/6J mice were injected bilaterally with the above-mentioned AAV vectors: at equivalent dose to (3.10^9^ vg/striatum).

### Processing of C57BL/6J mouse brain samples

For cell tropism analysis, 3-month-old female C57BL/6J mice were euthanized by intraperitoneal injection of euthasol. Mice were perfused intracardially with paraformaldehyde (PFA) 4% in 0.1 M Na_2_HPO_4_/NaH_2_PO_4_ buffer at pH 7.5 before dissection of the brains. Brains were post-fixed overnight in a PFA 4% solution at 4 °C. Brains were then cryoprotected by incubation in a 20% sucrose/0.1 M PBS solution for 24 h. Coronal brain Sects. (40 µm) were cut on a freezing microtome (Leica, Germany), collected serially in a cryoprotective solution, and stored at -20 °C until use. The sections analyzed were from the striatal region.

### Processing of mouse brain samples from R6/2 mice and WT littermates

For all experiments, 4-week-old male and female R6/2 mice and WT-littermates were rapidly euthanized by intraperitoneal injection of euthasol. Mice were perfused intracardially with ice-cold 0.1 M PBS before dissection of the brains. For immunohistochemistry, the left cerebral hemisphere was dissected and post-fixed in 4% PFA for 24 h and cryoprotected in 20% sucrose for 24 h. Coronal brain Sects. (40 µm) were cut on a freezing microtome (Thermo Scientific Microm HM 450, Germany), collected serially in a cryoprotective solution, and stored at -20 °C until use. The right hemisphere was dissected to dissociate the striatum for biochemical analysis (Western blot and ELISA) or biomolecular analysis (Vector copy number, VCN). Samples for biochemical analysis were then homogenized in lysis buffer (TBS, NaCl 150 mM and Triton 1%) containing phosphatase and protease inhibitors. After centrifugation (15 min, 13.000 rpm, 4 °C), the supernatant was collected, and the protein concentration was quantified by BCA assay (Thermo Fisher Scientific, Waltham, USA). Lysate were stored at − 80 °C. Sample for vector copy number were stored at − 80 °C until used.

For another cohort, the right cerebral hemisphere was dissected to extract the striatum which was collected in Eppendorf tubes, weighted empty, and filled for lipidomic analysis (Gas chromatography-mass spectrometry; GC–MS). These tubes were immediately frozen in dry-ice and stored at 80 °C until used. The left hemisphere was dissected, and striatum was extracted and collected in Eppendorf tubes for vector copy number studies while for RT-qPCR and RNA-seq studies, the tubes were snapped-frozen in liquid nitrogen.

### Primary antibodies

Details of antibodies used in western blot and immunohistochemical analyses can be found in Table 2**-** Primary antibodies used in Western blot (WB) and immunohistochemical (IHC) and Immunofluorescence (IF) analyses.

### Immunostaining in frozen free-floating brain slices from C57BL/6J mouse brain slices

The immunohistochemical procedure was initiated by quenching endogenous peroxidase through incubation of free-floating sections in hydrogen peroxide for 20 min at RT. After three washes, slices were blocked in PBS/0.2% Triton X-100 containing 5% normal goat serum (NGS, Gibco) for 1 h at RT. Sections were then incubated with the respective primary antibody diluted in PBS/0.2% Triton X-100 containing 3% NGS (Gibco), overnight at 4 °C (see Table 2). After three washes, sections were incubated with the corresponding biotinylated secondary antibody (1:500; Vector Laboratories Inc., CA, USA) diluted in PBS/0.1% Triton X-100 for 2 h, at RT. After three washes, bound antibodies were visualized by the ABC amplification system (1:250; Vectastain ABC kit, Vector Laboratories, West Grove, USA) and 3,3-diaminobenzidine tetrahydrochloride (peroxidase substrate kit, DAB, Vector Laboratories, CA, USA) as the substrate. Sections were dehydrated by immersing them through the following solutions: ethanol 50% (5 min immersion)**,** ethanol 70% (5 min immersions)**,** ethanol 95% (two immersions of 5 min each)**,** ethanol 100% (two immersions of 5 min each)**,** xylene (three immersions of 5 min each) and mounted on Superfrost® Plus (Thermo Fisher Scientific, USA) and coverslipped with Eukitt® (O. Kindler GmbH & CO, Freiburg, Germany).

For immunofluorescence, slices were washed with PBS 0.1 M, permeabilized in PBS-Triton 0.2% before blocking in PBS-Triton 0.2% containing 5% NGS (Gibco) for 1 h. Sections were then incubated with the respective primary antibodies, overnight at 4 °C. After three successive washes, brain slices were incubated for 2 h at room-temperature with fluorescent secondary Alexa Fluor-conjugated antibodies (1:500; Invitrogen, USA) lices were then mounted on Superfrost® Plus (Thermofisher Scientific, USA) and coverslipped with Fluormount (Sigma, France) medium.

### Immunostaining in frozen free-floating from R6/2 and wild-type littermates

For immunofluorescence, brain slices were washed with TBS 0.1 M, permeabilized and blocked in TBS-Triton 0.2% containing 10% NGS (Gibco) for 2 h. Sections were then incubated with the respective primary antibodies in TBS-Triton 0,2% containing 5% NGS, two days at 4 °C (see Table 2). After three successive washes, brain slices were incubated for 1 h and 30 min at room temperature with fluorescent secondary Alexa Fluor-conjugated antibodies (Invitrogen, USA). Slices were stained with Hoechst (1:20,000; Sigma, France), mounted in Prolong™ Gold antifade reagent and conserved at 4 °C.

### Imaging and cell tropism quantification in brain slices from C57BL/6J mice

To evaluate cell tropism of the various AAVrh10-HA-CYP46A1 constructs, images of HA/NeuN, HA/GFAP, HA/Iba1 and HA/Olig2 immunostained sections were acquired on a slide scanner, (Axio Scan Z.1, Zeiss, Germany) equipped with a Colibri illumination system (Colibri 2, Zeiss) and an Orca Flash 4.0 Hamamatsu.

The appropriate excitation filters were set as: 488 (green), 594 (red), and A Plan-Apochromat 10 × /0.45 objective for pre-focusing and a Plan-Apochromat 20 × /0.8 objective was used for fine focus image acquisition. Further analysis was performed using the *ImageJ* version 1.53c cell counter plug-in (NIH, Bethesda, USA). Three different regions of interest (ROI) were selected in the striatum, spanning three consecutives tissue sections per animal. In each ROI, the quantification process involved enumerating the number of cells expressing the transgene (NeuN, GFAP, Iba1 or Olig2), here referred to as CT + or CT- to simplify the explanation) and those solely expressing the transgene (HA).This categorization included counting HA + /CT- cells and double positive HA + /CT + cells, thereby providing the total count of HA + cells. The cumulative count across the three ROIs facilitated the determination of the total number of these three parameters. To address the question of the specific cell type affiliation of HA + cells, a ratio was calculated by dividing the count of HA + cells/CT + cells by the total number of HA + cells. This ratio was computed for each group and under each promoter's condition. A mean value was subsequently calculated across three adjacent tissue sections, providing a representative result for each experimental group and promoter type.

Representative images of immunostained sections were acquired by laser confocal microscopy (Leica SP8 model, Leica Microsystems, Germany). All images were acquired using a HC PL APO 40x/1.30 Oil CS2 objective, zoom: 4,75 and 12 for high magnification images. For each striatal coronal section, fields of view (512*pixel size^2^/µm^2^) were selected based on the HA-positive area.

### MSN area and Huntingtin aggregates’ imaging and quantification in brain slices from R6/2 mice

Images of immunostained sections (HA-DARPP-32, HA-EM48-NeuN or HA-EM48-S100b), were acquired by an inverted confocal laser scanning microscope (TCS SP8X, Leica Microsystems, Germany) equipped with a white light laser (470–670 nm, power measured at the objective entrance pupil: 0.16 mW for 488 nm) and a direct modulation laser 405 diode (0.16 mW, measured as described above). The acousto*-*optic tunable filter transmission rate was held constant for each wavelength used, and the HyD detectors operated in the photon-counting mode. All images were acquired using a HC PL APO 40x/1.30 Oil CS2 objective (pixel size: 0.284 µm; pixel dwell time: 400 Hz).

For each striatal coronal section, fields of view (1024*pixel size^2^/µm^2^) were selected based on the HA-positive area for coding constructs, and randomly selected for non-coding vectors. Stacks of 12 images, covering a total axial extent of 10.4 µm, were acquired per animal.

To assess the number of co-expressing NeuN + cells or S100β + cells with EM48 + positive cells, a custom macro was developed by the ICM Quant imaging facility (Paris, France). A 2D intensity manual threshold-based analysis was performed for each of the 12 stacks acquired per animal.

### Imaging of postmortem brain samples from HD subjects and healthy controls

Immunostained sections were acquired on a slide scanner, (Axio Scan Z.1, Zeiss, Germany) equipped with a Colibri illumination system (Colibri 2, Zeiss) and an Orca Flash 4.0 Hammatsu.

For brightfield immunostaining, a plan 10 × /0.45 objective for pre-focusing and a Plan-Apochromat 20 × /0.8 objective for fine focus image acquisition was used. Representative images were extracted using Zen blue 2.3 lite software (Zeiss, Germany). Briefly, manual threshold was applied for each staining (EM48, CYP46A1 and GFAP) and a zoom comprises between 180 and 250% was applied to images, following by a selected a region of interest (ROI).

#### Dendrite and spine analysis in Golgi-Cox-stained slices

#### Golgi-Cox staining

Brain hemispheres were incubated using the FD rapid Golgi stain kit (FD NeuroTechnologies, USA) according to the manufacturer's protocol. All procedures were performed under dark conditions. Coronal sections of 150 μm were cut with a vibrating microtome (Leica, VT1200S, Germany) while embedded in 2% agar prepared in 0.1 M PBS. Each section was mounted on an adhesive microscope slide pre-coated with 1% gelatin/0.1% chromalaun on both sides and stained according to the manufacturer’s protocol with the exception that AppliClear (AppliChem,Germany) was used instead of xylene. Finally, slices were mounted with PermountTM (Thermo Fisher Scientific,USA).

#### Imaging and image analysis of golgi-cox staining

Imaging of medium spiny dendritic branches within the striatum was performed (z-stack thickness of 0.5 μm) using an Axioplan 2 imaging microscope (Zeiss) equipped with a 63 × (N.A. 1.4) oil objective and a digital camera (AxioCam MRm, Zeiss, Germany). The number of spines was determined per micrometer of dendritic length using ImageJ (1.48v, National Instruments of Health, USA). Data were analyzed using GraphPad Prism (Version 5.01) software. Spine density is expressed as mean ± SEM. Differences between genotypes were detected with one-way ANOVA followed by Bonferroni’s post hoc test.

#### Western Blot in frozen striatal samples from R6/2 and wild-type littermates

Equal amount of total protein extracts (15 µg) were electrophoretically separated using 4–20% Criterion™ TGX Stain free™ Precast gels (Bio-rad, USA) and transferred to nitrocellulose membranes. Blocked membranes (5% non-fat dry milk in TBS-0.1% Tween-20) were incubated with the respective primary antibodies (see Table 2) overnight at 4 °C and washed three times with TBS–0.1% Tween-20 (TBS-T, Sigma, France) for 10 min. Membranes were then labelled with secondary IgG-HRP antibodies recognizing each corresponding primary antibody. After three washes with TBS-T, the membranes were incubated with ECL chemiluminescent reagent (Bio-rad, USA) according to the instructions of the supplier. Peroxidase activity was detected with Chemidoc™ Touch Imaging System (Bio-rad, USA) the optical densities were normalized with respect to a standard protein (GAPDH or vinculin).

### Animal behavior assessment

R6/2 mice and littermates were handled by the experimenter over three days at 5-week-old to minimize stress. Animals were acclimated in the resting room for 30 min before testing.

#### Rotarod

The rotarod protocol was slightly adapted from Menalled and colleagues [46] (R6/2 mice and littermates were tested over three consecutive days from 6 to 11 weeks of age. Each daily session included a 5-min training trial at a constant speed of 4 rpm on a rotarod apparatus (Bioseb, France). One hour later, the animals were tested for three consecutive accelerating trials of 5 min with the rotarod speed linearly increasing from (4 to 40 rpm over 300 s). The latency to fall from the rod (duration in seconds) was recorded for each trial. Mice remaining on the rod for more than 300 s were removed and their time scored, as 300 s. Data from the training trial (week 5) were not included.

### Cholesterol and oxysterol measurements using gas chromatography mass spectrometry (GC–MS)

Cholesterol and oxysterol analysis were adapted from the protocol described in [37] and from several ‘gold-standard’ methods to minimize the formation of autoxidation artefacts. During the extraction, samples were kept at 4 °C and /or under an inert atmosphere as much as possible. The mass spectrometer (Agilent 5975 inert XL) in series with the gas chromatography was set up for detection of positive ions. Detailed in Supplementary materials.

### DNA isolation and protein extraction

Genomic DNA isolation was performed with AllPrep® DNA/RNA/protein (Qiagen, Germany) according to the manufacturer's instructions. Following extraction, genomic DNA was stored at -20 °C, while RNA and protein samples were stored at -80 °C.

#### Analysis of vector genomes copies in frozen striatal tissue by qPCR

For each sample, 10 ng of DNA per well were used. Quantitative real-time PCR reactions were performed using LightCycler® 480 SYBR® Green I Master (Roche) according to manufacturer’s protocol, and reactions were run on LightCycler® 480 (Roche Diagnostics, Germany). The m*ADCK3* housekeeping gene was used to normalize the quantification of h*CYP46A1* levels. The list of primers used can be found in Table 3. The qPCR was performed according to the following program: initial denaturation step at 95 °C for 15 min followed by 40 cycles of amplification (Step 1: 95 °C for 10 s; Step 2: 65 °C for 20 s; Step 3: 72 °C for 20 s). Results (vector genome copy number per cell, VGC/cell) were expressed as n-fold differences in the transgene sequence copy number *(hCYP46A1)* relative to the m*ADCK3* gene copy (number of viral genome copy for 2N genome). Results were determined by the formula: N_*CYP46A1*_ = 2 ^2(ΔCt)^, where the Δ***C***_t_ value of the sample was determined by subtracting the ***C***_t_ value of the target gene from the ***C***_t_ value of the *mADCK3* gene.

**Table 3:** List of primers

Primer sequences	Forward	Reverse
*Abca1*	5’ CAACCCCTGCTTCCGTTATCCAA	5’ GAGAACAGGCGAGACACGATGGAC 3’
*Abca2*	5’ CAATATGCCAACTCCACGGTCAC 3’	5’ GGTCGCACTGGGTCGAACAA 3’
*Abcg1*	5’ TCTCCAATCTCGTCGCGTATCTGA 3’	5’ CAGATGCCACTTCCATGACAAAGTCT 3’
*ApoE*	5’ GTCACATTGCTGACAGGATGCCTA 3’	5’ GGGTTGGTTGCTTTGCCACTC 3’
*Dhcr7*	5’ AGCATTTGGGCCAAGACAC 3’	5’ AACCTGGCAGAAATCTGTGG 3’
*HmgcAred*	5’ TCTTGTGGAATGCCTTGTGA 3’	5’ TCTAGGACCAGCGACACACA 3’
*Hprt*	5’-TTGCTCGAGATGTCATGAAGGA-3’	5’-GCAGGTCAGCAAAGAACTTATAG-3’
*Human CYP46A1*	5’-GCAGCGGAGTCATAGACC-3’	5’-CAGCAGCATACTGGTCTCCA-3’
*Mouse Cyp46a1*	5’-TCCTCTCCTGTTCAGCACC-3’	5’-CAG CTTGGCCATGACAACT-3’
*Srebp1*	5’ GGTCCAGCAGGTCCCAGTTGT 3’	5’ CTGCAGTCTTCACGGTGGCTC 3’
*Srebp2*	5’ TGTTGACGCAGACAGCCAATG 3’	5’ GTTGCACCAGGACCGGGAC 3’
*mAdck3*	5’ CCACCTCTCCTATGGGCAGA 3’	5’ CCGGGCCTTTTCAATGTCT 3’

#### RNA extraction and Quantitative Real-Time Polymerase Chain Reaction

Samples were homogenized in QIAzol® reagent. RNA isolation was performed with miRNeasy Mini kit (Qiagen, Germany) according to manufacturer’s instructions. Reverse transcription was performed with RevertAid First Strand cDNA synthesis kit for RT-qPCR (Thermo Fisher, Lithuania), using 250 ng of RNA. Quantitative real-time PCR reactions were performed using LightCycler® 480 SYBR® Green I Master (Roche Diagnostics Gmbh, Germany) according to manufacturer’s protocol and run on LightCycler® 480 (Roche Diagnostics Gmbh, Germany). The expression of hypoxanthine guanine phosphoribosyltransferase 1 (*Hprt1)* transcript was used as an internal control for normalization. The cycle threshold values were calculated automatically by LightCycler® 480 SW 1.5 software with default parameters. The list of primers used can be found in Table 3. The qPCR was realized according to the following program, under 35 cycles: (step 1: 95 °C for 5 min; step 2: 95 °C for 15 s; step 3: 60 °C for 30 s; step 4: 72 °C for 30 s).

### RNA extraction, lightcycler real time polymerase chain reaction and RNA-sequencing study

12-weeks old R6/2 mice and littermates (*n* = 23), injected with AAV vectors, were perfused with ice cold PBS. The striatum was dissected, and immediately snap-frozen in liquid nitrogen, and stored at—80 °C until RNA isolation followed by reverse transcription, and quantitative PCR reactions.

Total RNA was extracted following the protocol of RNeasy Mini Kit (QIAGEN). After extraction, total RNA was qualified with AGILENT tapeStation 2200. mRNA library preparation was realized following manufacturer’s recommendations (KAPA mRNA HyperPrep Kit from ROCHE). Final samples pooled library prep were sequenced on ILLUMINA Novaseq 6000 platform, corresponding to 2 × 25 Millions of reads per sample after demultiplexing. Ingenuity Pathway Analysis (IPA Qiagen, Courtaboeuf, France) was used differentially expressed transcripts in an experimental data set to derive enriched cellular functions and to predict upstream regulators. Statistical significance (p-overlap) of predictions was determined by comparing the number of differentially expressed transcripts in an experimental dataset with the total number of transcripts linked to a function or regulator. An activation Z‐score was derived from known interactions to predict the activated or inhibited state of putative transcriptional regulators [47]. Pathway and function enrichment were assessed with Fisher's exact test in Ingenuity analyses (IPA). RNASeq gene expression data and raw fastq files are stored on the GEO repository (www.ncbi.nlm.nih.gov/geo/) under accession number: GSE220224. The preparation and sequencing of mRNA libraries was performed by the iGenSeq core facility, at the *Institut du Cerveau* (ICM) at the Pitié-Salpêtrière Hospital (Paris, France).

### Statistical analyses

Statistical analyses were performed with GraphPad Prism 8.4.3. All data in this report are represented as mean ± SEM. Statistical analyses were performed for data obtained from 12 week-old male and female R6/2 mice and age-matched controls, with experiments conducted on samples from the striatal region (for biomolecular, biochemical, and histological samples. For each the following analysis normality and homoscedasticity were assessed: DARPP-32 (analysis of MSN area), spine density, EM48 staining and triple staining (analysis of Huntingtin aggregates in different cell types), lipidomic studies (GC–MS), Vector copy number, RT-qPCR, ELISA and Western Blot studies as well as rotarod test. If both of these conditions were respected, parametric tests were applied: one-way ANOVA followed by a Dunnett’s post-hoc test. For the rotarod test, a two-way ANOVA was used followed by a Dunnett’s post-hoc test with time and treatment as independent factors. In the case either, normality test or homoscedasticity was not respected, we used non-parametric test: Kruskal–Wallis followed by Dunn’s *post-hoc* test. Regarding lipidomic studies (GC–MS), we performed multiple unpaired t-tests corrected for multiple comparisons using the Holm-Sidak method. Finally, spine density was analyzed using a one-way ANOVA followed by a Bonferroni *post-hoc* test. All other pertinent information, including sample size and specific statistical test used, can be found in the figure legends or are labelled within the figures.

## Results

### CYP46A1 is decreased in neurons and re-expressed in striatal glial cells from HD patients

**Fig. 1:**
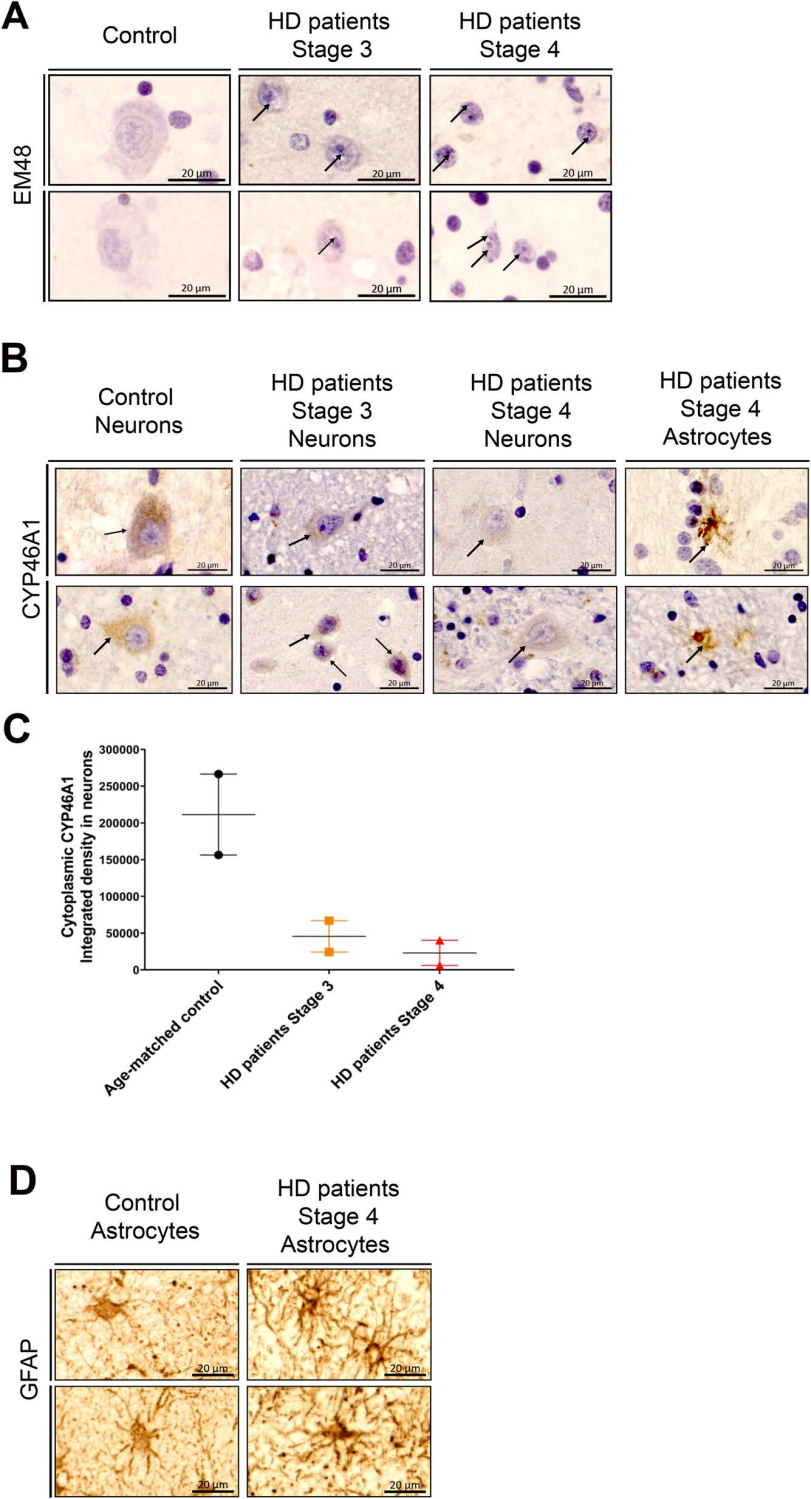
CYP46A1 is re-expressed in astrocytes of HD patients. **A** Representative immunohistochemically labelled brain sections of putamen (Anti-HTT immunostaining using the EM48 antibody, counterstained with Hematoxylin) from HD patients (n = 4) and age-matched controls (n = 2) revealed nuclear and cytosolic mutant Huntingtin aggregates in neurons (black arrows) but not in control individuals. Scale bar: 20 μm. **B** Anti-CYP46A1 immunostaining counterstained with Hematoxylin showing intense and diffuse cytoplasmic CYP46A1 immunoreactivity in control neurons (n = 2) while it was decreased in neurons from HD patients at stage 3 and 4 (n = 4), stage 4 HD patients also exhibited re-localization of CYP46A1 in astrocytic cell-like soma and dendrites (n = 2) (black bold arrows). Scale bar: 20 μm. **C** Quantification of CYP46A1 cytoplasmic intensity staining in HD patients at Vonsattel stage 3 (n = 2), at Vonsattel stage 4 (n = 2) and age-matched controls (n = 2). **D** Diaminobenzidine (DAB)-directed immunostaining for GFAP in the striatum of control individuals (n = 2) and HD patients (n = 4): hypertrophic soma of astrocytes was detected in HD patients relatively to those from control individuals. Scale bar 20 μm

The human postmortem striatum regions highly affected in HD from 5 different cases with confirmed HD and three age-matched control individuals with no signs of neurodegeneration were analyzed by immunohistochemistry to detect the presence of misfolded HTT. For this, the mEM48 antibody, specific for the expanded polyQ tract [7, 48], was used; subjects carrying the Huntingtin mutation showed EM48-positive inclusions in the nucleus and cytosol of striatal cells; as expected, no inclusions were found in healthy controls (Fig. 1A).

The impairment of brain cholesterol metabolism is detrimental in HD [20, 23]. CYP46A1 is physiologically expressed in neurons [38] and is decreased in the putamen of HD subjects [36], but the localization of CYP46A1 expression was never studied in HD subjects.

High intensity CYP46A1 staining was found in the cytosol of neurons from control individuals (n = 2), whereas in HD subjects (*n* = 5) CYP46A1 staining in striatal neurons was weaker as assessed by quantitative measurement in two HD subjects at Vonsattel stage 3 and two HD subjects at Vonsattel stage 4. Interestingly, in the putamen of HD subjects, at Vonstattel stage 4, CYP46A1 expression was occasionally found in star-shaped cells: astrocyte-like glia (Fig. 1B and 1C). This ectopic expression was not observed in the Vonsattel stage 3 putamen. GFAP immunostaining in the putamen of HD subjects at stage 4 revealed reactive astrocytes as seen by the hypertrophic processes compared to age matched control astrocytes (Fig. 1D). These data suggest that CYP46A1 is decreased in neurons and expressed in reactive glial cells, possibly reactive astrocytes, in human HD postmortem putamen at late stage.

### Expression characterization after injection of HA-CYP46A1 vectors with, neuronal and astrocytic promoters, or ubiquitous promoter

**Fig. 2:**
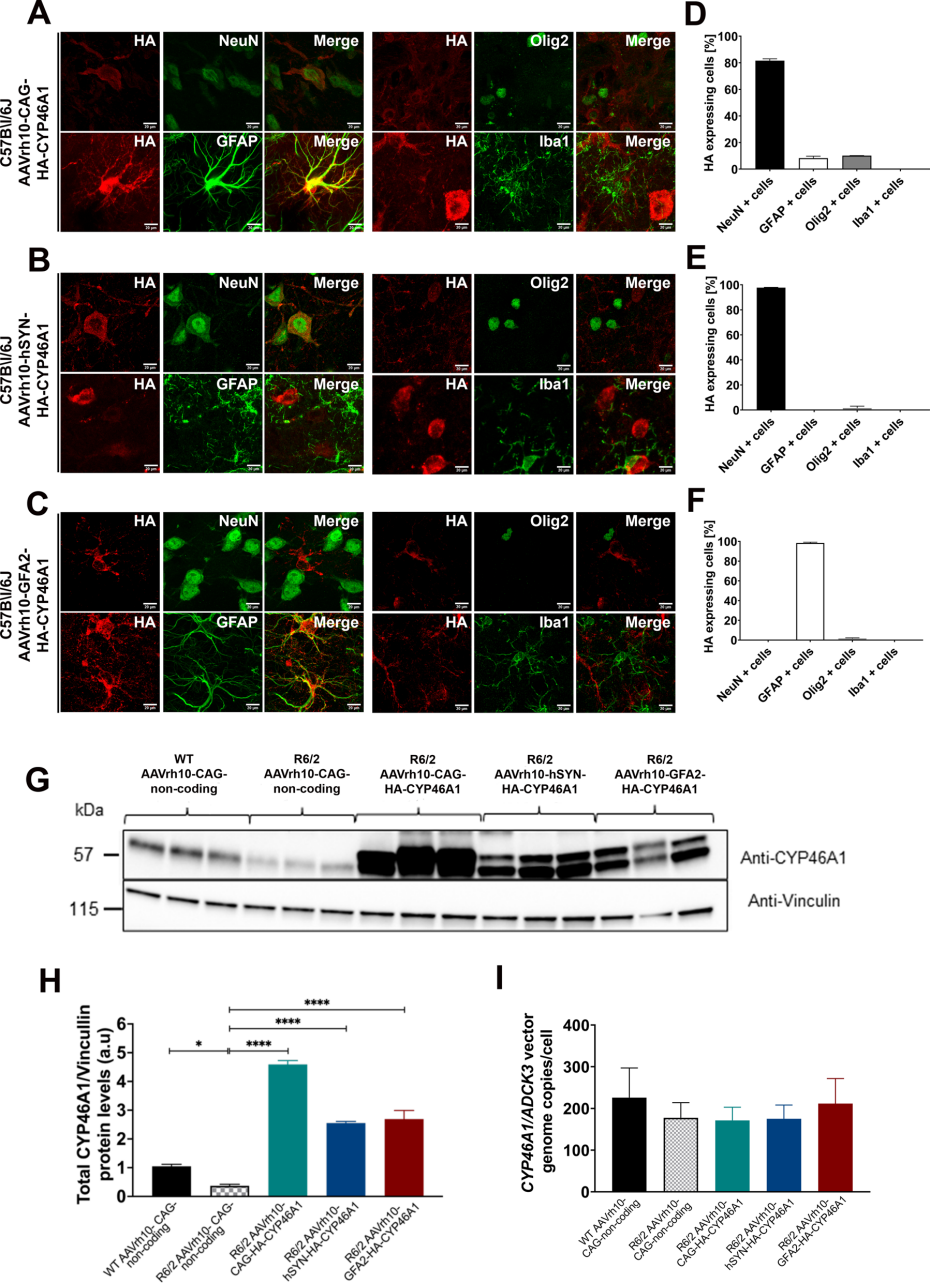
Evaluation of cellular tropism based on neuronal and glial promoter selectivity and transduction efficacy after delivery of AAVrh10 vectors coding for HA-CYP46A1 in mouse striatum. **A–C** Representative laser confocal microscopy images of double immunofluorescence between HA (red) and several cell-type specific markers [NeuN for neurons (green), GFAP for astrocytes (green) and Iba1 for microglia (green), and Olig 2 for oligodendrocytes (green)] in coronal brain slices from C57BL/6J, 3 weeks post-injection. Scale bar: 20 μm. **D** Quantification of cellular tropism after AAVrh10-CAG-HA-CYP46A1 (n = 3) showing that among HA + cells, 82% are NeuN + cells, 10% are Olig2 + and 8% GFAP + cells. No detectable HA-CYP46A1 immunoreactivity was detected in microglia (Iba1). **E** Quantification of cellular tropism after AAVrh10-hSYN-HA-CYP46A1 (n = 3) showing that among all HA + cells, 98.4% are NeuN + cells and 1.6% are Olig2 + ; no immunoreactivity was detected in both Iba1 + and GFAP + cells. **F** Quantification of cellular tropism after AAVrh10-GFA2-HA-CYP46A1 injection (n = 3) showing that among all HA + cells, 98.5% are GFAP + cells and 1.5% are Olig2 + ; no immunoreactivity was detected in both NeuN + and Iba1 + cells. Data are represented as the mean ± S.E.M. **G–H** Representative western blot of total CYP46A1 levels in striatal extracts from 12-week-old R6/2 mice and injected with the different constructs and age-matched WT-littermates (8 weeks after injection). For optical densitometry quantification signal intensities were normalized to vinculin protein, used as loading control. Data are represented as the mean ± S.E.M (n = 3/group). Statistical analysis was performed using One-way ANOVA followed by Dunnett *post-hoc* test: * P < 0.05; **** P < 0.0001 relatively to R6/2 non-coding as mean group control. **I** Quantification of vector genome copy number per cell assessed by qPCR in the striatum of R6/2 mice injected with the different constructs showing that no statistically significant differences were found between groups. Data are represented as the mean ± S.E.M (n = 3–6/group). Statistical analysis was performed using One-way ANOVA followed by Dunnett *post-hoc* test, with R6/2 non-coding as mean control

To determine the molecular and functional effect of CYP46A1 in astrocytes or neurons, we first characterized the HA immunoreactivity area after AAVrh10-mediated HA-CYP46A1 delivery with the different promoters (Supplementary Fig. 1A), in the striatum of 3-week-old C57BL/6J female mice. AAVrh10-CAG-HA-CYP46A1, AAVrh10-hSYN-HA-CYP46A1 and AAVrh10-GFA2-HA-CYP46A1 at equivalent doses (2µL, 3E9 vg/striatum) allowed comparable volume of distribution of HA-CYP46A1 in the striatum of C57BL6/J mice (45%, 39% and 42%, respectively, n = 3 mice per group) (Supplementary Fig. 1B and C), 3 weeks after injection. As expected, no HA-CYP46A1 detection was observed following injection with the control non-coding vector (n = 3). We then investigated the targeting of different cell types using constructs coding for HA-CYP46A1 using specific GFA2 and hSYN promoters. To evaluate cell type-specific tropism, double immunofluorescence was employed between HA and the different cell type specific markers (Olig2 for oligodendrocytes, GFAP for astrocytes, NeuN for neurons and Iba1 for microglia (Fig. 2A-F)*.* The injection of AAVrh10-CAG-HA-CYP46A1 allowed major HA detection in neurons (82%), astrocytes (8%), oligodendrocytes (10%) but not in microglia (n = 3) (Fig. 2A and D)*.* The hSYN-promoter restricted HA-CYP46A1 expression to neurons (98.4%) and oligodendrocytes (1.6%) but not in astrocytes nor in microglia (Fig. 2 B and E)*.* Conversely, the GFA2 promoter allowed HA-CYP46A1 detection essentially in astrocytes (98.5%) and only 1.5% in oligodendrocytes, but not in neurons nor in microglia (Fig. 2 C and F)*.* Finally, we assessed the impact of CYP46A1 targeting of astrocytes, neurons or both cell types on neuronal integrity. We showed that for equivalent injected vector dose, overall preservation of the neuronal nuclei marker (NeuN) (Supplementary Fig. 1B and C) and the medium spiny neurons marker (DARPP-32) (Supplementary Fig. 1B and D) were observed.

We next injected these vectors into the striatum of 4-week-old male and female R6/2 mice and quantified HA-CYP46A1 protein expression by western blot 8 weeks after injection (n = 3 mice per group). To assess the total levels of CYP46A1 expression, we quantified both murine CYP46A1 (lower band) and transgenic human CYP46A1 (upper band). Total CYP46A1 protein levels were promoter-dependent: GFA2 and hSYN- promoters enabled comparative CYP46A1 overexpression (sevenfold) higher compared to mice receiving control non-coding CYP46A1 (stuffer). CAG-mediated CYP46A1 expression levels were 12-fold higher compared to endogenous CYP46A1 protein levels in mice receiving control vectors (Fig. 2G–H). Western-blot probed with anti-HA antibody confirmed that the non-coding vector (negative control) did not allow detection of HA-CYP46A1 product when compared to AAVrh10-CAG-HA-CYP46A1, thus confirming immunohistochemistry data (Supplementary Fig. 2A and B). Importantly, vector copy number per cell, measured by qPCR, was comparable across the different groups (Fig. 2I).

### Cholesterol homeostasis’ regulation by CYP46A1 overexpression in neurons or astrocytes in R6/2 mice

**Fig. 3:**
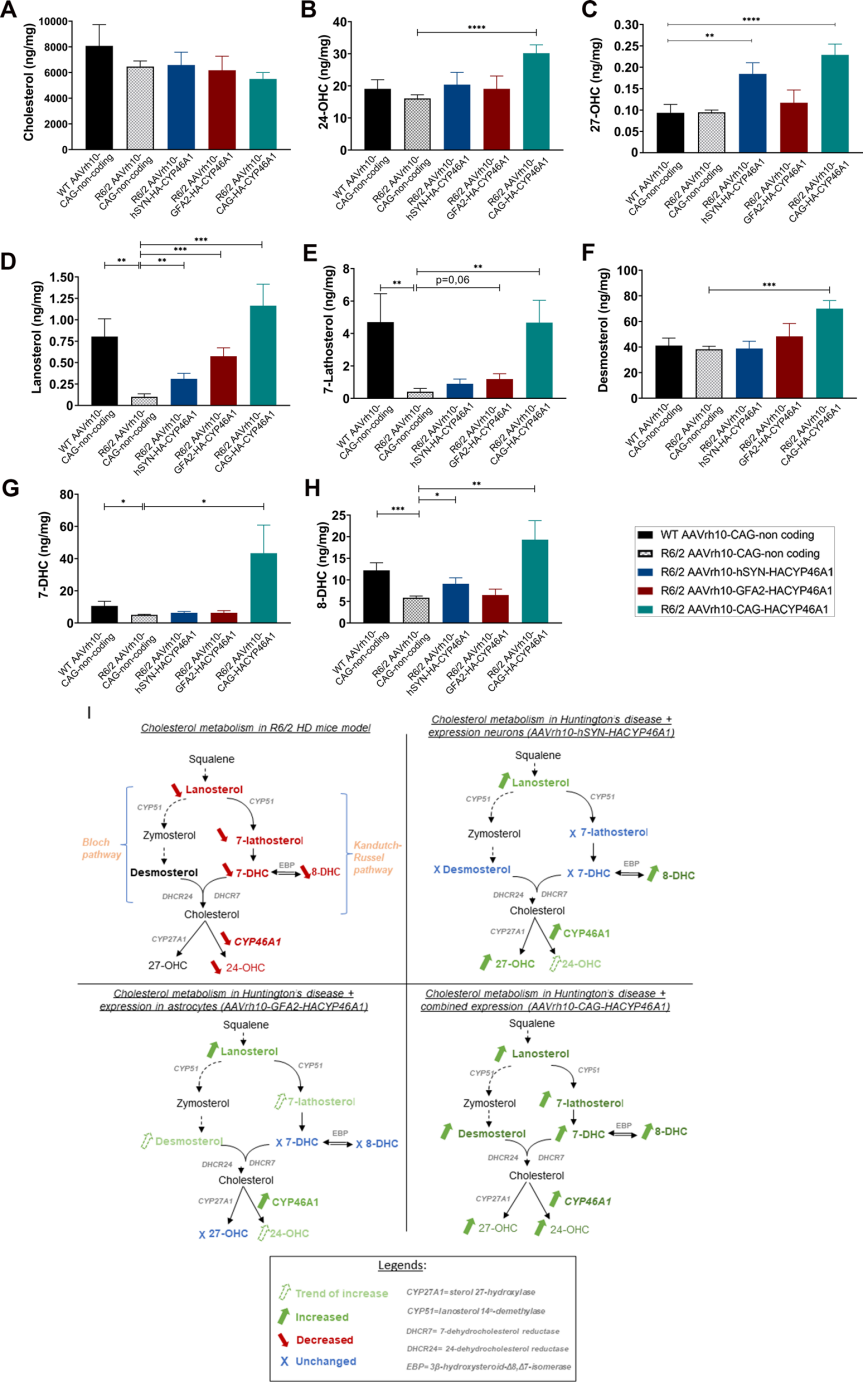
Regulation of cholesterol homeostasis by CYP46A1 overexpression in striatal neurons or astrocytes of R6/2 mice, 8 weeks after striatal injection. Quantification of Cholesterol **A**, 24S-OH-Cholesterol **B**, 27-OHC **C**, Lanosterol **D**, 7-Lathosterol **E**, Desmosterol **F**, 7-Dehydrocholesterol (7-DHC) **G** and 8-Dehydrocholesterol (8-DHC) **H** in striatal tissue from 12 weeks-old (i.e., 8 weeks post-injection) WT and R6/2 mice injected with non-coding vector or AAVrh10-HA-CYP46A1 mediated by CAG, hSYN, GFA2 promoters. Results are represented as the mean ± SEM, (n = 7–10 independent mice). Statistical analyses: Multiple t-test * P < 0.05; **P < 0.01; *** *P < *0.001; **** P < 0.0001. **I** Schematic representations of Cholesterol precursors, Cholesterol, and oxysterol deficit in Huntington’s disease (upper left panel) and the impact CYP46A1 overexpression under either CAG promoter (upper right panel), hSYN promoter (lower left panel) and GFA2 (lower right panel). Legends are detailed at the bottom

In HD, cholesterol synthesis, transport and degradation of cholesterol are impaired [20, 23, 25]. To assess the functionality of the various vectors we performed quantitative measurements of sterols and oxysterols by GC–MS in 12-week-old R6/2 striatal extracts. We first compared cholesterol, 24S-OHC, and 27-OHC levels between littermates (n = 7), and R6/2 mice injected with the different vectors into the striatum (Fig. 3A–C). Total cholesterol levels were overall comparable among the different groups. A non-statistically significant increase in the levels of 24-OHC was detected when CYP46A1 was expressed by the mean of the hSYN (n = 10) or GFA2 promoters (n = 10) but were significantly increased by 87.5% in the striatum of R6/2 mice injected with AAVrh10-CAG-HA-CYP46A1 (****P < 0.0001) (n = 10). Levels of 27-OHC were increased in R6/2 mice injected with AAVrh10-hSYN-HA-CYP46A1 (**P < 0.01) relatively to control non-coding injected-mice while AAVrh10-GFA2-HA-CYP46A1 did not impact 27-OHC levels. Mice injected with AAVrh10-CAG-HA-CYP46A1 (****P < 0.0001) had significantly increased levels of 27-OHC levels.

Lanosterol, the first intermediate sterol of the biosynthesis pathway, as well as lathosterol and desmosterol, intermediate precursors of cholesterol belonging to the Kandutsch-Russell and Bloch pathways, respectively, were analyzed. As previously described [23], lanosterol levels were decreased in the striatum of R6/2 mice relatively to WT littermates (Fig. 3D). The levels of lathosterol were significantly lowered in R6/2 mice [49] (Fig. 3E), while the desmosterol content remained unchanged (Fig. 3F) relatively to WT-littermates, both injected with non-coding control. CYP46A1 overexpression in R6/2 mice, mediated by the use of hSYN and GFA2 promoters, significantly increased the levels of lanosterol compared to control R6/2 mice (***P < 0.001; ****P < 0.0001 respectively). An increase for 7-lathosterol (*P* = 0.06) that was not statistically significant was also observed with CYP46A1 GFA2-mediated overexpression, while CYP46A1 hSYN-mediated overexpression in R6/2 mice had no impact on 7-lathosterol levels. Though, 7-dehydrocholesterol (7-DHC) levels remained unchanged in the hSYN and GFA2 groups, although the former showed an increase of 8-dehydrocholesterol (8-DHC) (**P < 0.01) compared to control R6/2 mice. CYP46A1 expression in R6/2 mice directed by hSYN promoter did not impact desmosterol levels while a non-statistically significant increase could be detected with the use of GFA2 relatively to control R6/2.

Of note, AAVrh10-CAG-mediated overexpression of CYP46A1 significantly increased of the levels of several all cholesterol precursors lanosterol (****P < 0.0001), desmosterol (****P < 0.0001), 7-lathosterol (**P < 0.01), 7-dehydrocholesterol (*P < 0.05), and 8-dehydrocholesterol (**P < 0.01) relatively to control R6/2 mice.

Mouse *Cyp46a1* mRNA levels were decreased inl 12 week-old male and female R6/2 receiving non-coding CYP46A1 compared to WT receiving the same control vector (Supplementary Fig. 3A). However, the use of different promoters to overexpress CYP46A1 in R6/2 striatum did not impact the endogenous mouse *Cyp46a1* transcript. The non-coding construct enables expression of hCYP46A1 mRNA levels however a frameshift mutation in the hCYP46A1 cDNA sequence, generates a stop codon stop that prevents translation by the ribosomal machinery (Supplementary Fig. 2C, Supplementary 3A-B).

Human Cyp46a1 mRNA levels were not significantly different among groups, although WT and R6/2 non-coding groups, as well as, R6/2 mice injected with CAG-CYP46A1 have higher mRNA levels than R6/2 mice injected with hSYN-CYP46A1 and GFA2-CYP46A1 (Supplementary Fig. 3B). As a ubiquitous promoter, CAG drives stronger expression than cell-type specific promoters such as hSYN and GFA2.

We next studied the impact of human CYP46A1 overexpression at the transcription levels on genes involved in the regulation of cholesterol homeostasis, known to be dysregulated in HD. CYP46A1 overexpression in R6/2 mice with the different constructs did not impact these transcripts (Supplementary Fig. 3C-J).

CYP46A1 expression in astrocytes or neurons activates the cholesterol synthesis pathway. Combined targeting using CAG promoter increased all cholesterol precursors, as well as the oxysterol 24-OHC levels while, maintaining cholesterol level, confirming that AAV-CAG-CYP46A1 restores brain cholesterol metabolism [36, 37].

### CYP46A1 overexpression in astrocytes improves mHTT aggregate clearance in neurons and astrocytes

**Fig. 4:**
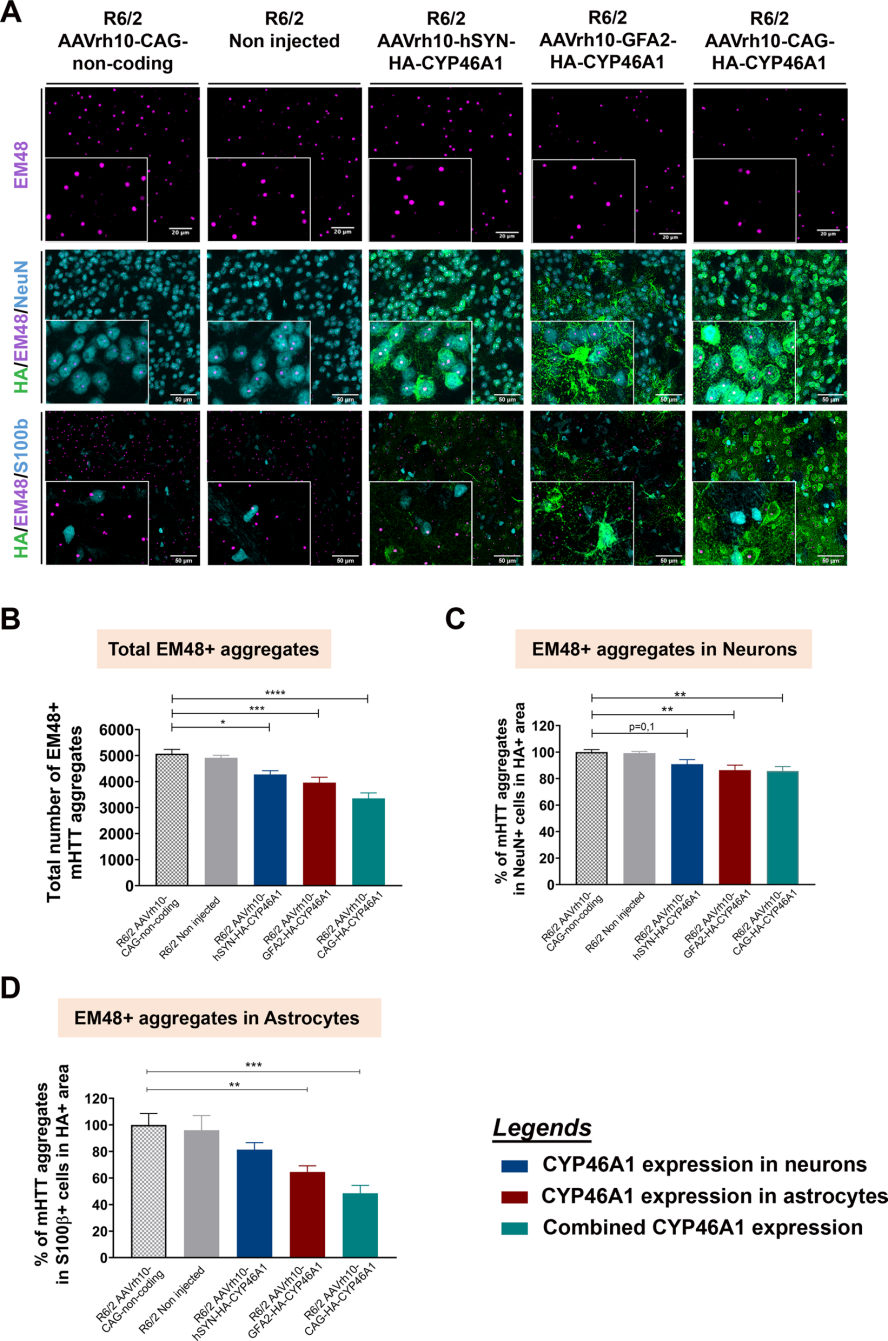
Overexpression of CYP46A1 in striatal astrocytes or neurons from R6/2 mice decreases the number of Huntingtin aggregates in a cell and non-cell autonomous effect, 8 weeks after striatal injection. **A** Upper panel: representative images of mHTT aggregates in the R6/2 striatum (EM48 + , violet dots) in HA + area (green) among the different groups, injected with the different AAV constructs(n = 10–12/group). Middle panel: representative images of mHTT aggregates (EM48 + , violet dots) in HA + area (green) among the different R6/2 groups in NeuN + cells (cyan) (n = 5–7/group). Lower panel: representative images of mHTT aggregates (EM48 + , violet dots) in HA + area (green) among the different R6/2 groups in S100β + cells (cyan) (n = 5–7/group). **B** Quantified data showing the percentage of reduction of the total amount of mHTT aggregates in neurons following AAV-CYP46A1 delivery mediated by CAG, hSYN and GFA2 promoters in HA + positive area. No differences were detected between non-injected R6/2 and R6/2 mice injected with non-coding vector. Data are represented as the mean ± S.E.M. Statistical analysis: one-way ANOVA followed by Dunnett’s post-hoc test (**P < 0.01 R6/2 AAVrh10-CAG-HA-CYP46A1 vs R6/2 AAVrh10-non-coding; **P < 0.01 R6/2 AAVrh10-GFA2-HA-CYP46A1 vs R6/2 AAVrh10-non-coding). **C** Quantified data showing the percentage reduction of the total amount of mHTT aggregates in neurons following CYP46A1 delivery mediated by CAG and GFA2 promoters. No differences were observed between non-injected R6/2 and R6/2 mice injected with non-coding vector and in R6/2 receiving AAVrh10-hSYN-CYP46A1 (P = 0.098). Data are represented as the mean ± S.E.M. Statistical analysis: one-way ANOVA followed by Dunnett’s post-hoc test (** P < 0.01 R6/2 AAVrh10-CAG-HA-CYP46A1 vs R6/2 AAVrh10-non-coding; **P < 0.01 R6/2 AAVrh10-GFA2-HA-CYP46A1 vs R6/2 AAVrh10-non-coding). **D** Quantified data showing the percentage reduction of the total amount of mHTT aggregates in astrocytes following CYP46A1 delivery mediated by CAG and GFA2 promoters. No differences were observed between non-injected R6/2 and R6/2 mice injected with non-coding vector and in R6/2 receiving AAVrh10-hSYN-HA-CYP46A1. Data are represented as the mean ± S.E.M. Statistical analysis: one-way ANOVA followed by Dunnett’s post-hoc test (***P < 0.001 R6/2 AAVrh10-CAG-HA-CYP46A1 vs R6/2 AAVrh10-non-coding; ** P < 0.01 R6/2 AAVrh10-GFA2-HA-CYP46A1 vs R6/2 AAVrh10-non-coding)

The accumulation of mHTT aggregates, a hallmark of HD, is detected both in neurons and astrocytes in 12-week-old male and female R6/2 mouse striatum [8]. We compared the impact of cell-specific CYP46A1 overexpression on the aggregates content in neurons and in astrocytes. Therefore, a triple immunofluorescence staining with the EM48 antibody, detecting mHTT aggregates in combination with HA and NeuN (neuronal marker) or S100β (astrocytic marker) was performed (Fig. 4A).

Following HA-CYP46A1 delivery in astrocytes via the GFA2 promoter, a 22.3% reduction in mHTT aggregates was observed across all cells % (n = 11 mice per group, 12 slices per animal) (Fig. 4B) (Table 4). Specifically, a reduction of 13.8% was observed in neurons (NeuN + cells) (Fig. 4C) while a reduction of 31.9% in astrocytes (S100β + cells) was detected (Fig. 4D). When CYP46A1 was delivered in neurons (AAVrh10-hSYN-HA-CYP46A1 injected group, the reduction was slightly lower- about 16.2% of total aggregates (n = 10 mice per group, 12 slices per animal). The number of aggregates in neurons was reduced by 9.5% in neurons (NeuN + cells), while the number of aggregates in astrocytes was reduced by 18.7% in astrocytes (S100β + cells) (n = 5 mice per group; 12 slices per animal).

**Table 4:** Summary of mHTT aggregates reduction following AAV-HA-CYP46A1 with various promoters

Reduction of mHTT aggregates	AAVrh10-hSYN-HA-CYP46A1	AAVrh10-GFA2-HA-CYP46A1	AAVrh10-CAG-HA-CYP46A1
Overall reduction	^a^16.2%	^c^22.3%	^d^34%
NeuN + cells	9.5%	^b^13.8%	^b^14.6%
S100b + cells	18.7%	^b^31.9%	^c^51.5%

^a^Significance: *P < *0.05

^b^Significance: *P < *0.01

^c^Significance: *P < *0.001

^d^Significance: *P < *0.0001

Combined delivery using the CAG promoter (AAVrh10-CAG-HA-CYP46A1) resulted in the most significant aggregate reduction: reduction by 34% in the HA + striatal area (Fig. 4B), with a 14.6% reduction in neurons (NeuN + cells) (Fig. 4C) and 51.5% in astrocytes (S100β + cells) (Fig. 4D) relative to R6/2 controls (non-coding). A negligible difference in the total number of aggregates was found between non-injected and non-coding groups (Fig. 4B, C and D).

Altogether, both neuronal and astrocytes HA-CYP46A1 expression after AAV-HA-CYP46A1 striatal delivery, allow major reduction of mHTT aggregates in astrocytes. Interestingly, CYP46A1 expression in astrocytes targeting is more efficient in reducing aggregates in both neurons and astrocytes than specific neuronal targeting. The use of the CAG promoter that combines expression in both neurons and astrocytes, together with higher levels of expression using the CAG promoter, allows optimal overall correction of mHTT aggregates.

#### CYP46A1 overexpression in astrocytes or neurons mitigates neuronal and spine density atrophy

**Fig. 5:**
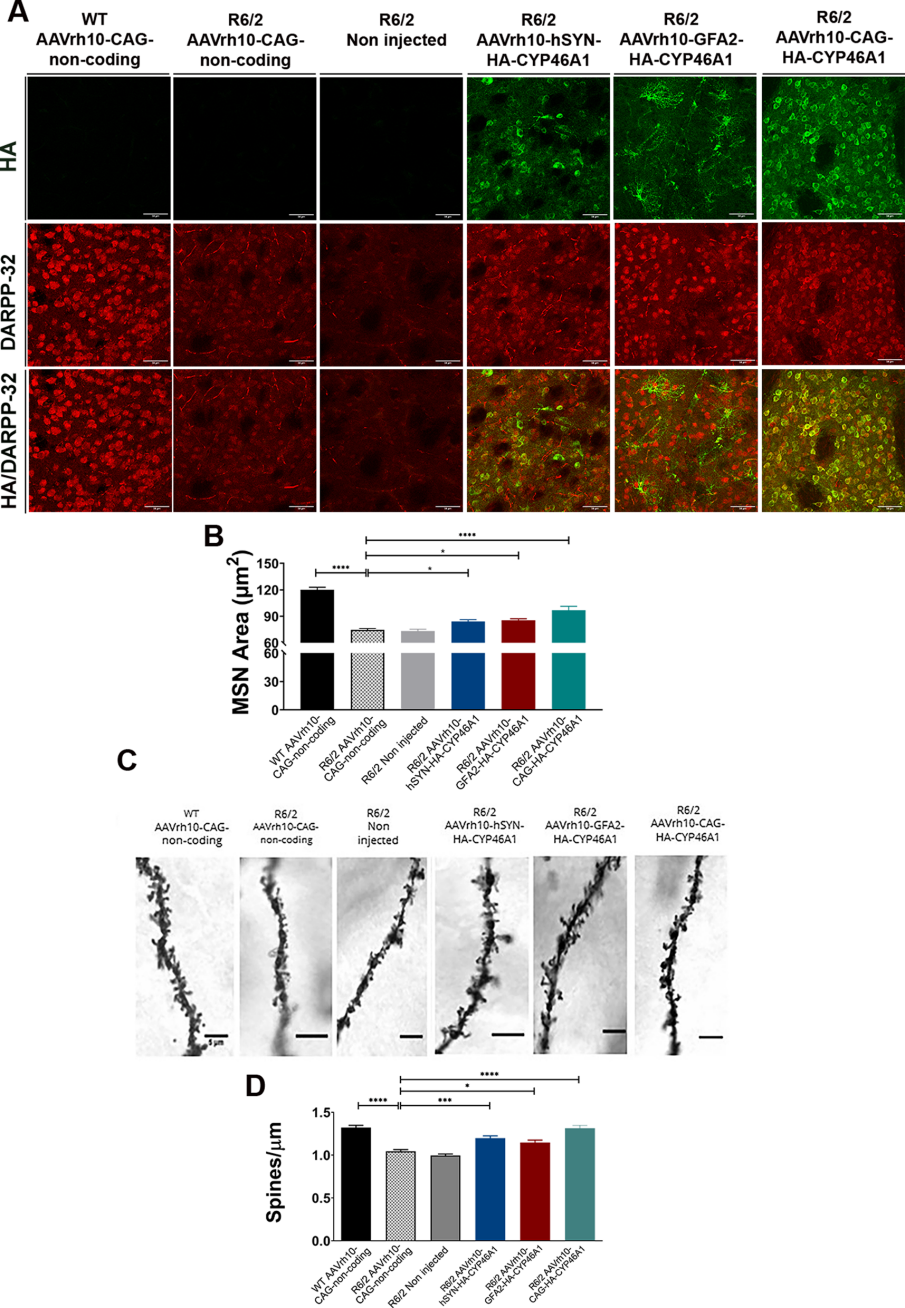
MSN atrophy and reduced spine density are improved upon CYP46A1 delivery in l neurons and/or astrocytes from R6/2 mice in a cell and non-cell-autonomous dependent mechanism, 8 weeks after injection. **A** Representative laser confocal microscopy images of the double immunostaining between DARPP-32, a marker of medium spiny neurons (red) in HA + area (green), in brain slices from R6/2 mice and WT littermates, injected with the different AAV constructs. **B** The quantification of MSN area is expressed in mm^2^. Data are represented as the mean ± S.E.M. Statistical analysis: one-way ANOVA followed by Dunnett’s post-hoc test [****P < 0.0001 WT AAVrh10-CAG-non-coding (n = 7) vs R6/2 AAVrh10-CAG-non-coding (n = 6); ****P < 0.0001 R6/2 AAVrh10-CAG-HA-CYP46A1 (n = 5) vs R6/2 AAVrh10-CAG-non-coding; *P < 0.05 R6/2 AAVrh10-hSYN-HA-CYP46A1 (n = 5) and R6/2 AArh10-GFA2-HA-CYP46A1 (n = 6) vs R6/2 AAVrh10-CAG-non-coding (n = 6); *P < 0.05 R6/2 AAVrh10-CAG-HA-CYP46A1 (n = 5) vs R6/2 AAVrh10-hSYN-HA-CYP46A1 (n = 5); ***P < 0.001 R6/2 AAVrh10-CAG-HA-CYP46A1 (n = 5) vs R6/2 AAVrh10-GFA2-HA-CYP46A1 (n = 6)]. **C** Images of Golgi-Cox-stained medium spiny dendrites in brain slices from R6/2 mice and age-matched WT littermates injected with the different AAV constructs. Scale bar = 5 mm. **D** Spine number was reduced in AAVrh10-CAG-non-coding injected mice (N = 4/n = 29) compared to WT AAVrh10-CAG-non-coding injected mice (N = 4/n = 32) (****P < 0.0001), and then improved upon overexpression of HA-CYP46A1 driven by the CAG (N = 4/n = 25), GFA2 (N = 4/n = 31) and hSYN (N = 4/n = 31) promoters in the R6/2 mouse striatum (***P < 0.0001, * P < 0.05 and *P* = 0.0001, respectively). Data are represented as mean ± SEM. one-way ANOVA followed by Bonferroni’s post-hoc test. “N” represents the number of animals and “n” the number of dendrites

A key pathological hallmark of HD pathology is the atrophy of medium spiny neurons (MSN), a marker of neurodegeneration [50]. In this study, 12-week-old male and female R6/2 mice injected with a control non-coding vector were compared to WT littermates injected with the same vector. As expected, R6/2 mice exhibited significant neuronal atrophy, as indicated by reduced MSN area assessed by DARPP-32 immunostaining. This atrophy was significantly improved in neurons from R6/2 in the AAVrh10-hSYN-HA-CYP46A1 (*P < 0.05) and with AAVrh10-GFA2-HA-CYP46A1 (*P < 0.05) compared to control R6/2 mice (Fig. 5A–B). Enhanced mitigation of MSN atrophy was observed with mice injected with AAVrh10-CAG-HA-CYP46A1 (****P < 0.0001). Since dendritic spine degeneration, synaptic loss and altered neurotransmission occur early in HD mouse models. [51] we next evaluated whether CYP46A1 restoration in neurons and/or astrocytes was reflected at the functional neuronal network level [52–54]. Spine density of medium spiny neurons in the striatum was assessed on dendritic segments stained with the Golgi-cox method (Fig. 5C–D). We found that spine density was significantly reduced in both R6/2 mice injected with “non-coding vector” (n = 4 mice, 34 dendrites analyzed) and non-injected R6/2 mice (n = 4 mice, 29 dendrites analyzed), compared to WT controls (n = 4 mice, 32 dendrites) (*P < *0.0001, one-way ANOVA followed by Bonferroni’s post hoc test).

This spine phenotype was partially rescued due to CYP46A1 overexpression controlled by GFA2 (n = 4 mice, 31 dendrites) and hSYN promoters (n = 4 mice and 31 dendrites) (*P < *0.05 and *P* = 0.0001 respectively) as spine density was significantly increased. (Fig. 5C–D). The spine phenotype was fully rescued when CYP46A1 overexpression was driven by means of the CAG promoter as spine density in AAVrh10-CAG-HA-CYP46A1 injected R6/2 mice (n = 4 mice with 25 dendrites analyzed) showed no significant difference compared to WT controls (n = 4 mice with 32 dendrites analyzed) and was significantly increased in comparison to control R6/2 mice controls (*P < *0.0001) and R6/2 non-injected mice (*P < *0.0001).

In summary, we provide evidence that CYP46A1 expression restricted in neurons or in astrocytes can partially mitigate MSN atrophy and to improve spine density in a severe mouse model of HD. Further, we provide evidence that when CYP46A1 expression is driven by the CAG promoter, optimal neuroprotection is achieved, and HD spine phenotype is fully rescued.

### CYP46A1 overexpression in astrocytes corrects motor imbalance in R6/2 mice better than in neurons

**Fig. 6:**
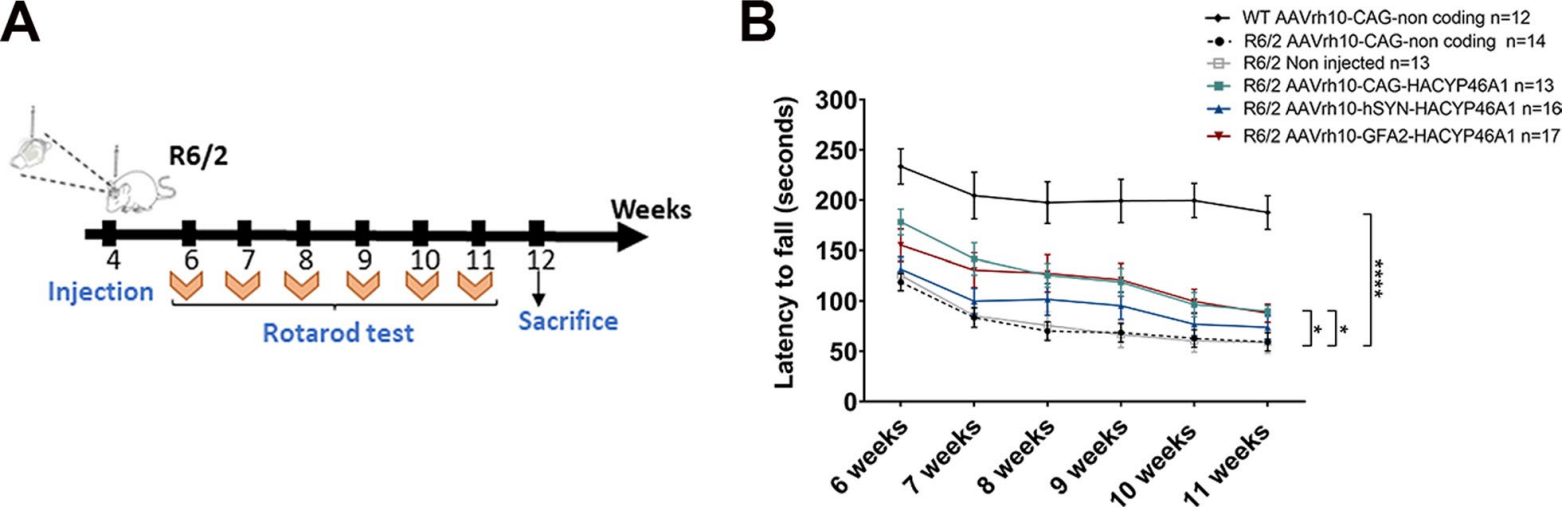
Overexpression of CYP46A1 in striatal astrocytes and neurons improves motor abilities in R6/2 mice. **A** Experimental set-up for striatal injection of CYP46A1 and time frame of behavioral tests performed. **B** Rotarod performances of wild-type littermates, non-injected R6/2 mice and R6/2 mice injected with control non-coding vector or the different AAV-CYP46A1 coding constructs were assessed by the latency to fall (expressed in seconds) from 6 to 11 weeks (measured for each group). Blinded-randomization of the group was maintained throughout the test. Data are represented as the mean ± SEM. Statistical analysis: Two-way ANOVA followed by Dunnett’s post-hoc test with time and treatment as independent factors [(**** *P < *0.0001: WT-type non-coding (n = 12) vs R6/2 non-coding (n = 14); *P < 0.05: R6/2 CAG (n = 13) vs R6/2 non-coding (n = 14) and R6/2 GFA2 (n = 17) vs R6/2 non-coding (n = 14)]

R6/2 mice recapitulate many features found in HD subjects, in particular, motor impairment, appearing before 6 weeks of age [46, 55, 56]. To assess CYP46A1 beneficial effects, all constructs enabling HA-CYP46A1 expression mediated by the different promoters, were bilaterally injected into the striatum of 4-weeks-old R6/2 mice. Behavioral testing was then conducted from 6 to 12 weeks of age (Fig. 6A).

Motor imbalance in R6/2 mice and WT-littermates was assessed using the accelerating rotarod between 6 and 11 weeks of age. As expected, R6/2 mice injected with control non-coding vector (AAVrh10-CAG-non-coding (stuffer) exhibited a significant reduction in the latency to fall compared to WT littermates (****P < 0.0001) (Fig. 6B). No significant differences were observed between non-injected R6/2 mice and those injected with the control non-coding vector, confirming that the non-coding vector had no impact on behavioral phenotype.

CYP46A1 overexpression in astrocytes from R6/2 mice (AAVrh10-GFA2-HA-CYP46A1) led to a significant improvement in motor balance from week 6 to week 11 (increased by 49% by week 11) (Fig. 6B), while a trend was observed in R6/2 mice treated with AAVrh10-hSYN-HA-CYP46A1 compared to R6/2 injected with control non-coding vector, it was not statistically significant (increased by 25% by week 11). In contrast, combined neuronal and astrocytic expression of CYP46A1 in R6/2 injected with AAVrh10-CAG-HA-CYP46A1 resulted in a significant improvement in motor balance from week 6 to week 11 (increased by 52% by week 11) compared to R6/2 injected with control non-coding vector.

Finally, whatever the promoter used, CYP46A1 overexpression did not impact the gain weight of R6/2 mice (Supplementary Fig. 4), indicating that the observed motor improvements are not attributable to body weight differences. Overall, these data suggest that astrocytes-specific CYP46A1 overexpression significantly improves motor abilities in R6/2 mice and is more effective than neuronal-specific expression. Moreover, combined targeting using the CAG promoter further enhances the therapeutic benefits, offering an optimized strategy for mitigating motor dysfunction.

### Overexpression of CYP46A1 in neurons and astrocytes modulates specific cellular pathways

Targeting CYP46A1 expression specifically in astrocytes proved to be more efficient at clearing aggregates and improving motor performance compared to neuronal targeting. To further investigate the molecular effects of CYP46A1 overexpression, in neurons and/or in astrocytes, we performed RNA sequencing (RNA-seq) in the striatum of 12-week-old male and female R6/2 mice injected with AAV-CYP46A1 constructs driven by astrocyte-specific (GFA2), neuron-specific (hSYN) or combined (CAG) promoters. These were) compared to R6/2 or WT mice injected with the control non-coding vector (AAVrh10-CAG-non-coding). Transcriptional differences were evaluated with principal component analysis (PCA) realized by CLC genomics software. PCA shows an HD samples aggregation from controls, with the genotype as the main effect (Supplementary Fig. 5).

**Fig. 7:**
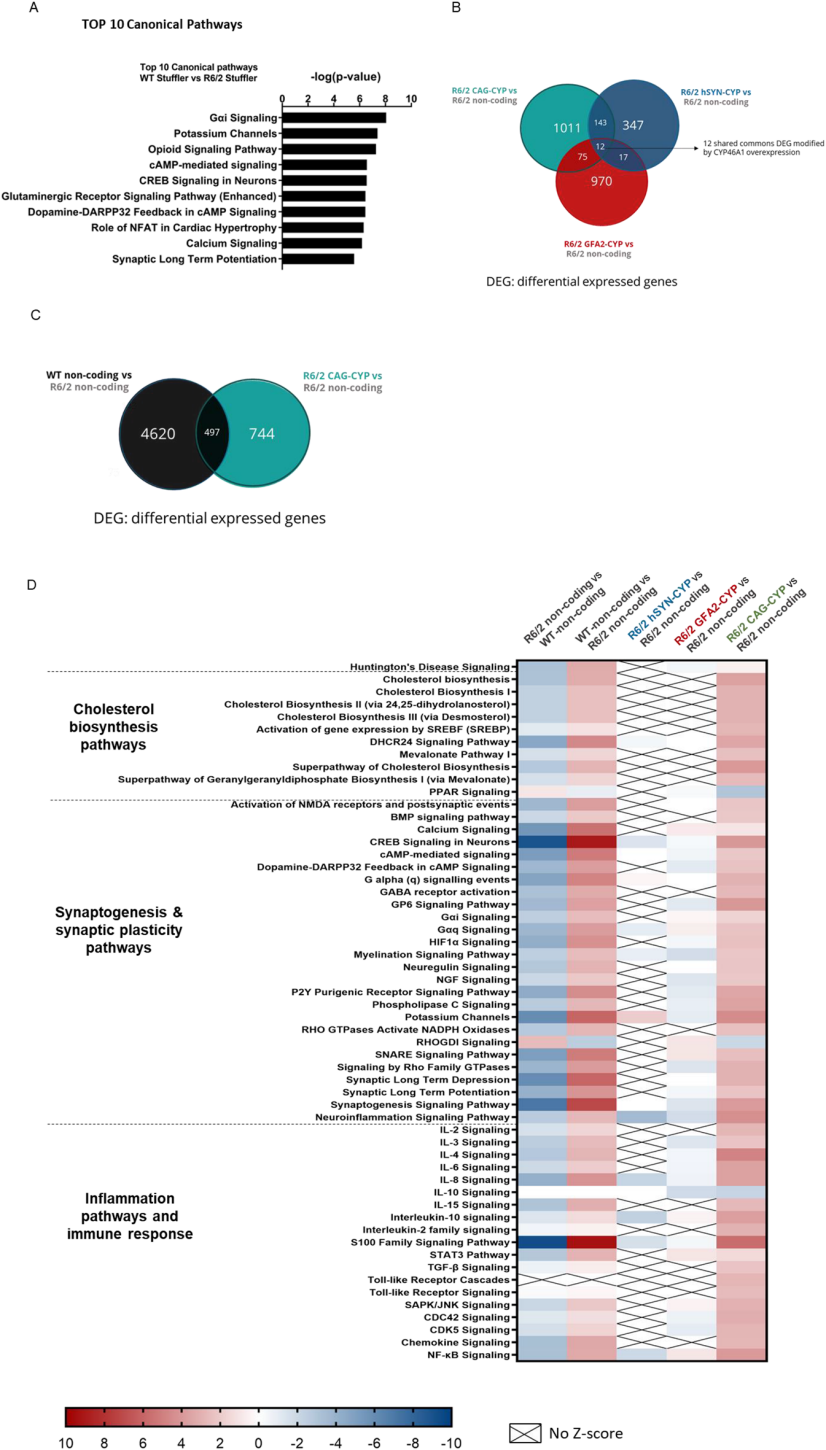
Striatal overexpression of CYP46A1 driven by mean of glial and neuronal promoters modifies major transcriptome pathways, including cholesterol biosynthesis, inflammation cascades, synaptogenesis, and synaptic plasticity pathways, 8 weeks after injection. **A** Top 10 IPA canonical pathways WT-CAG-non-coding (n = 4) vs R6/2 CAG-non-coding injected mice (n = 4). **B** The number of DEGs overlap between R6/2 CAG-non-coding (n = 4) vs R6/2 AAVrh10-CAG-HA-CYP46A1 (green) (n = 5) and R6/2-CAG-non-coding (n = 4) vs R6/2 AAVrh10-GFA2-HA-CYP46A1 (red) (n = 5) and R6/2-CAG-non-coding (n = 4) vs R6/2 AAVrh10-hSYN-HA-CYP46A1 (blue) (n = 4). ** C** The number of DEGs overlap between WT-CAG-non-coding (n = 4) vs R6/2 CAG-non-coding (n = 4) and vs R6/2 AAVrh10-CAG-HA-CYP46A1 (green) (n = 5) vs R6/2 CAG-non-coding (n = 4) ** D** Pathways are significantly altered (cut off: *P < *0.05; z-score = 2 (absolute value)) in R6/2 CAG-non-coding compared to WT-CAG-non-coding, 8 weeks after injection; and R6/2 AAVrh10-CAG-HA-CYP46A1, AAVrh10-GFA2-HA-CYP46A1, or R6/2 AAVrh10-hSYN-HA-CYP46A1 compared to R6/2 CAG-non-coding. Cholesterol pathways are highlighted in green. The IPA z-score indicates if the pathway is predicted to be inhibited (blue), activated (red) or activation or inhibition cannot be predicted (grey)

Our results identified altered pathways in WT littermates compared to WT R6/2 mice, in particular, synaptogenesis and synaptic long-term potentiation (Fig. 7A; Supplementary Fig. 6A).

Overexpression of CYP46A1 under neuron-specific and astrocyte-specific promoters led to 347 and 970 differentially expressed genes (DEGs) respectively, compared to R6/2 non-coding controls. In the CAG-CYP46A1 group, 1011 DEGs were identified, when compared to R6/2 non-coding group, including 12 commonly differentially expressed genes with hSYN-CYP46A1 and GFA2-CYP46A1 groups. Moreover, 497 DEGs were found when WT and R6/2 groups were compared (Fig. 6B–C; Supplementary Fig. 6B).

Differentially expressed genes were clustered to sets of genes that are linked to specific pathways [57]. We used the statistical approach based on the definition of the z-score, to deduct the activation state (“increased" or “decreased”) of pathways. Most pathways were predicted to still be inhibited or unchanged in GFA2-HA-CYP46A1 and hSYN-HA-CYP46A1 groups compared to non-coding injected group (Fig. 6C; Supplementary Fig. 6C). Of note, WT non-coding vs R6/2 non-coding comparison exposes the transcriptome aspects from the WT control stand-point, while the reverse comparison (R6/2 Stuffler vs WT stuffler) refers to the HD condition. Interestingly, most pathways that were predicted to be downregulated (negative z-score) in HD condition (R6/2 non-coding vs WT non-coding comparison), were shown to be upregulated in the CAG-HA-CYP46A1 group (Supplementary Fig. 6C).

Pathways predicted to be significantly activated in the CAG-HA-CYP46A1 group showed a positive z-score for such as synaptogenesis (Z score = 3.9) and synaptic long-term potentiation (Z-score = 2.31), cholesterol biosynthesis (Z score = 3.64) and especially cholesterol metabolism via desmosterol, and the mevalonate pathway (Z-score = 3 and 2.45, respectively). Interestingly, other pathways such as Toll-like Receptor Signaling (Z score = 2.65), Interleukin-6 (IL-6) and IL-8 Signaling (Z-score = 3.64), and neuroinflammation signaling (Z-score = 4.32) were also predicted to be activated. The detailed list of targeted genes regulated by CYP46A1 within these selected pathways is presented in Supplementary Fig. 6D. Nearly all genes involved in the cholesterol metabolism pathway were significantly modified by CAG-HA-CYP46A1, confirming the findings obtained in lipidomic studies. Similar results were reported in Kacher et al., [37] where other genes were also modified by CYP46A1: *Ube2l6* and *Psmb9* (ubiquitin), *Cxcr3* (immune response), *Acp1, Lamp-5* (autophagy-related) (Supplementary Fig. 6D). In conclusion, while hSYN- and GFA2-mediated CYP46A1 overexpression did not exhibit major impact within the transcriptome pathways, CAG-mediated expression of CYP46A1 positively and widely impacted transcriptome pathways related to synaptogenesis, synaptic plasticity, and inflammation pathways in the striatum of R6/2 mice.

### CYP46A1 overexpression in astrocytes decreases striatal levels of TGF-β1 in R6/2 mice

Given the transcriptomic finding indicating activation of several interleukin pathways, we conducted an ELISA assay to measure the levels of several interleukins in striatal tissue of 12 week-old R6/6 mice, aiming to better complement findings regarding inflammatory response following CYP46A1 overexpression. Several of these cytokines including Tumor Necrosis Factor α (TNF-α), IL-1β, IL-6, IL-12, and IL-10 are altered in HD mouse models and human post-mortem tissue [58, 59]. We found equivalent levels of striatal Transforming Growth Factor (TGF-β1), IL-6, IL-1β, between R6/2 mice and WT-littermates injected with non-coding vector. Strikingly, TGF-β1 levels were overall drastically reduced following CYP46A1 overexpression with GFA2 promoters (*P < 0.05), while a slight decrease, that was not statistically significant, was observed for mice injected with hSYN-HA-CYP46A1 (*P < *0.08) compared to R6/2 mice injected with non-coding vectors (Supplementary Fig. 7A). However, no significant effect was seen on IL-1β (Supplementary Fig. 7B) or IL-6 (Supplementary Fig. 7C) in these groups.CYP46A1 overexpression under the CAG promoter led to a pronounced decrease of TGF-β1 levels. The levels of IL-1β (Supplementary Fig. 7B) and interleukin-6 (IL-6) (Supplementary Fig. 7C) were comparable to those observed in WT-littermates. Although a twofold increase in IL-1β was observed after CAG-HA-CYP46A1 injection compared to R6/2 non-coding controls, this difference was not statistically significant (P = 0.07). Similarly, IL-6 levels showed a 1.5-fold increase, but variability within the group 1.5-fold but was not statistically different, possibly due to the high variability precluded statistical significance. No significant changes were detected for IL-2, IL-4, IL-10 and IL-12p70 levels across treatment groups (Supplementary Fig. 7D-G). In summary, our data show that astrocytic CYP46A1 overexpression significantly reduced TGF-β1 levels in the striatum of R6/2 mice, while its effects on other cytokines remain limited.

## Discussion

Astrocytes play supportive biological roles in neuronal performance. They provide metabolic and trophic support, antioxidant defense, and regulate synaptic transmission and synaptic plasticity [15]. However, their contribution in HD pathogenesis still remains incompletely characterized. Growing evidence suggests that in HD, the expression of mHTT in astrocytes impairs astrocytic functions contributing to neuronal dysfunction and cell death, or exacerbating the harmful effect of mHTT in neurons [8, 9, 12, 13]. While mHTT accumulates in astrocytes, the number of aggregates is lower compared to that observed in neurons [11, 60, 61]. Reduced glutamate uptake, altered K^+^ buffering, impaired regulation of blood flow, reduced synthesis of gliotransmitters, trophic factors [61–65], and cholesterol [25, 51], are the main consequences of defective astrocyte function in HD. Cholesterol cannot cross the BBB and brain cholesterol is produced in situ, mostly in astrocytes, in adults [16]. Brain cholesterol metabolism is maintained by the neuron-specific enzyme CYP46A1, which allows for brain cholesterol efflux, since cholesterol cannot cross the BBB. In HD, cholesterol synthesis by astrocytes is reduced and associated with reduced transport to neurons via ApoE. *Cyp46a1* transcripts are downregulated in post-mortem human HD striatum, as well as in R6/2 mice and in knock-in mouse models of HD [66]. Consistently, CYP46A1 protein levels are decreased in the striatum of both HD subjects and animal models [36, 37, 39]. Disrupted brain cholesterol homeostasis occurring in HD highlights the complex interplay between neurons and astrocytes. Here, we demonstrate in the striatum of HD subjects (Vonsattel Stage 4) [67], CYP46A1 is expressed in occasional astrocytes-like cells, while overall CYP46A1 expression is decreased in neurons. This suggests that astrocytes-like cells might contribute to CYP46A1 production to compensate for reduced neuronal CYP46A1 expression. Exposure of primary rat cultures of hippocampal and cortical astrocytes to IL-6 was shown to induce a remarkable increase of CYP46A1 only in activated GFAP-positive astrocytes, suggesting that CYP46A1 expression is triggered in reactive astrocytes in responses to proinflammatory signals [68]. Interestingly, relocalized expression of CYP46A1 in astrocytes has also been reported in several neurological conditions such as after traumatic brain injury [69], in a model of Multiple Sclerosis (MS) [69] and in the brain of patients with Alzheimer’s disease (AD) [70–72], possibly reflecting an adaptive response to altered cholesterol metabolism [71].

Cholesterol is essential to neuronal function and survival. The outgrowth of neurites requires an important supply of cholesterol for axonal guidance and synapse/dendrite formation [73, 74]. Cholesterol is required for sustained synaptic activity by generation of neurotransmitter vesicles [75]. Therefore, it is not surprising that changes in cholesterol content and thus, in cholesterol metabolism (cholesterol synthesis, transport and uptake), may contribute to neuropathological processes in severe neurodegenerative disorders such as Alzheimer’s disease [76, 77], Huntington’s disease [20] and Parkinson’s disease [78]. In HD, it is clearly demonstrated that reduced cholesterol synthesis impairs synapse maturation, neurotransmitter vesicle generation and synaptic activity [25] with major consequences on HD pathophysiology.

This study aimed to elucidate the specific role of astrocytes in Huntington's disease, focusing on cholesterol pathway dysfunction and its impact on disease phenotype. We used an AAV-based delivery approach to target CYP46A1 expression in mouse striatum. We first confirmed that targeting CYP46A1 expression in astrocytes, using a GFA2 [79] promoter, in the striatum of wild-type and R6/2 mice is safe and efficiently improves impaired cholesterol metabolism.

To assess the cell-type-specific therapeutic effects of CYP46A1, we selectively delivered the gene to neurons (using AAVrh10-hSYN-HA-CYP46A1) or astrocytes (using AAVrh10-GFA2-HA-CYP46A1) in the striatum of R6/2 mice. Importantly, overall comparable levels of CYP46A1 expression were achieved when expression was driven in neurons (hSYN promoter) or in astrocytes (GFA2 promoter), thus enabling this comparative analysis.

mHTT accumulates in neurons and astrocytes, and mHTT aggregates are detected in the nucleus and the cytoplasm of both cell types [9, 11, 60, 80]. The consequences of mHTT expression in astrocytes have been broadly demonstrated [8, 20, 61, 63, 64, 81]. Indeed, astrocytic mHTT expression is sufficient to cause neuropathology and behavioral impairments, while specific mHTT ablation in astrocytes can slow down disease progression and restore physiological function [12]. Total HTT protein levels are similar in both cell types in the wild-type mouse [11, 82]. However, in HD mouse models, striatal neurons display more mHTT aggregates than astrocytes [11, 60], and during the course of the disease, the size of nuclear mHTT aggregates increases in neurons but not in astrocytes [11]. In R6/2 mice, we observed that CYP46A1 expression in neurons (AAVrh10-hSYN-HA-CYP46A1) led to a significant reduction of mHTT aggregates in neurons, but not in astrocytes. In contrast, astrocytic expression of CYP46A1 (AAVrh10-GFA2-HA-CYP46A1) resulted in a significant reduction of mHTT aggregates in both astrocytes and neurons. Moreover, the greater overall reduction of aggregates following astrocytic expression points to potentially enhanced capacity of astrocytes to promote mHTT clearance compared to neurons [83, 84].

Astrocytic overexpression of CYP46A1 in R6/2 mice was sufficient to improve reduce mutant huntingtin aggregates in both astrocytes and medium-spiny neurons (MSNs), supporting a non–cell-autonomous protective effect. This is consistent with previous reports showing that reactive astrocytes can mitigate protein aggregation and neurotoxicity [85–87].

Reduction of mHTT aggregates in HD mouse models after AAV-CYP46A1 delivery is a consequence of the restoration of the mevalonate pathway of cholesterol synthesis by CYP46A1 [36, 37]. evidenced by upregulation of enzymes such as *Srebf2*, *Hmgcr*, and *Dhcr24*—and increased levels of cholesterol precursors (lanosterol, demosterol). A non-statistical increase in desmosterol production is observed with astrocytic CYP46A1 targeting, suggesting potential activation of the Bloch pathway of cholesterol synthesis [88, 89].

Sterol precursors produced by CYP46A1 expression were shown to activate cellular proteostasis and clearance of misfolded proteins [37, 90, 91], and are expected to contribute to the decrease of mHTT [92]. Lanosterol can promote protein aggregate clearance through enhanced autophagy and co-chaperone activation [90, 91].

Indeed, treatment with lanosterol or desmosterol could reduce mHTT aggregates accumulation in primary cultures of striatal neurons transfected with mHTT [37]. Lanosterol was also shown to confer neuroprotection in a MPTP (1-methyl-4-phenyl-1,2,3,6-tetrahydropyridine) model of Parkinson’s disease [93] and to activate autophagic clearance of misfolded proteins. The role of the mevalonate pathway on autophagic flux was also associated with protein farnesylation and geranylgeranylation [94]. While we did not directly measure autophagy and proteasome activity, RNA-seq revealed upregulation of key ubiquitin–proteasome system components (e.g., *Ube2l6, Psmb9*) and autophagy-related transcripts. These changes align with earlier studies showing CYP46A1 enhances proteostasis and neuronal function [37]. Notably, these effects occurred despite cell-type-specific targeting, supporting a non–cell-autonomous mechanism, potentially mediated by cholesterol-dependent signaling. Beyond the activation of the autophagic clearance of mHtt, the neuroprotection observed following CYP46A1 overexpression is consistent with the role of the mevalonate pathway in promoting synaptic and neurite development [95] and with prior reports of increased synaptic markers following CYP46A1 overexpression [96, 97].

An important consideration is whether the neuroprotective effects observed in HD models stem from CYP46A1 protein activity on the mevalonate pathway activation or from its direct enzymatic product, 24-hydroxycholesterol (24-OHC). 24-OHC could contribute to the neuroprotective effects observed through potential activation of GTPases geranylgeranylation secondary [95, 97], reduced TGF-β1 levels and modulation of LXR [36, 98]. The concomitant changes in CYP46A1 and 24-OHC levels—such as those seen after AAV-mediated knockdown [99]—make it difficult to isolate their individual contributions. It is likely that both act in concert, through LXR signaling and isoprenoid-mediated remodeling, to support neuronal resilience in HD.

To further investigate the non–cell-autonomous effects of astrocytic CYP46A1, we analyzed striatal transcriptomic profiles following cell-type-specific overexpression. Transcriptional impairment is a major component in HD progression [100], and one the earliest events of the HD phenotype [101, 102]. This has been well described in R6/2 mice [103, 104] re astrocyte transcriptomic studies reported downregulation of genes involved in the biosynthesis pathways of cholesterol in both R6/2 and zQ175 mice [105, 106]. Astrocytic CYP46A1 upregulated LDL receptor expression (z-score: 2.83), suggesting enhanced neuronal uptake of sterol metabolites, while combined expression more strongly activated cholesterol efflux genes such as ApoE (z-score: 3.82) and ABCA1 (z-score: 2.08). These results support the idea that astrocytes modulate the local cholesterol environment in ways that impact neuronal function. Future fluorescence-activated cell sorting (FACS)-based RNA-seq studies could further clarify cell-type-specific responses. Additionally, assessing cholesterol dynamics in membrane microdomains could help link sterol changes to aggregation. Our RNA-seq analysis after CAG-mediated CYP46A1 expression shows predicted activation of the cholesterol biosynthesis pathway via the Bloch pathway and desmosterol production, and is confirmed by increased lanosterol and desmosterol in the striatum of AAVrh10-CAG-HA-CYP46A1 treated R6/2 mice. The activation of the cholesterol pathway is confirmed by the increased levels of cholesterol-related molecules such as Dhcr-24, SREBPF-2 which showed a twofold increase (see Supplementary Fig. 6). SREBP2 is a sensor of cholesterol levels, which can regulate the transcription of mevalonate pathway genes [107]. Interestingly, our transcriptomic analysis predicts SREBP2 to be activated with CAG mediated CYP46A1 expression (z-score = 2.99) and GFA2-mediated expression (z-score = 2.19), suggesting that activation of the mevalonate pathway by CYP46A1 occurred though SREBP2. This result is in line with a previous study showing that nuclear expression of Srebp2, Hmgcr and Dhcr24 were increased by CYP46A1 in zQ175 HD mouse model. [37, 108]

Interleukin pathways are altered in HD [109] and our results show that their transcriptomic profile is modified by CYP46A1 expression. Strikingly, we show that TGF-β1 level is strongly reduced following CYP46A1 overexpression in astrocytes. TGF-β is implicated in temporal neurogenesis and neural stem cells potency in the CNS [110] and its impairment is involved in HD pathogenesis [111–113]. TGF-β1 and its signaling are increased in induced pluripotent stem cell derived-neural stem cells from HD patients [114] and in striatal cell line expressing mHTT. [113]

In R6/2 mice, as well as in transgenic HD rat model, elevated levels of TGF-β signaling are observed and induce quiescence of neuronal stem cells, impairing neuronal differentiation and disrupting neurogenesis [112]. Increased TGF-β signaling in HD animal models elevates the expression of the mutant form of huntingtin, potentially contributing to neurodegeneration [115, 116]. TGF-β1 is predominantly expressed by astrocytes, and its levels gradually increase with Vonsattel stages in HD post-mortem striatum [117].

Here, we evidenced a significant decrease of TGF-β1 levels in R6/2 mouse striatum when CYP46A1 was overexpressed in astrocytes while only a trend was noted when CYP46A1 was overexpressed in neurons. This reduction of TGF-β1 might contribute to the mitigation of MSN atrophy and to the improvement of spine density and highlights the contribution of astrocytes in the therapeutic effect of AAV-CP46A1 gene therapy. This is consistent with the effect of TGF-β signaling inhibition observed in ALS mice [118]. Motor performance improved more significantly when CYP46A1 expression was targeted in astrocytes relatively to neurons. Rotarod test of R6/2 mice injected with AAVrh10-GFA2-HA-CYP46A1 was significantly improved, while only a trend for recovery was observed after AAVrh10-hSYN-HA-CYP46A1 injection. Our data confirm the role of astrocytes in mitigating the HD phenotype, since astrocytic transduction in HTT-lowering approaches is required to rescue behavioral phenotypes in HD mice, while neuronal targeting alone was insufficient to mitigate HD progression [14].

MSN atrophy and reduced spine density are hallmarks of neurodegeneration in HD and have been reported in R6/2 mice [50, 52, 119]. The activation of the Mevalonate pathway and CYP46A1 were shown to regulate both dendritic and axonal outgrowth in vitro [95].

In rat cortical neurons, CYP46A1 overexpression and activation of the mevalonate pathway increase synaptic markers and enhance dendritic protrusion density and outgrowth. These effects are mediated through activation of Trk receptors and geranylgeranylation of GGTases [95, 97]. Similar results were found in CYP46A1 transgenic mice, where increased levels of synaptic proteins in the hippocampus were associated with enhanced spatial memory retention in aged animals [96]. In line with this, AAV-CYP46A1 delivery through the use of CAG promoter mitigated MSN atrophy in R6/2 and ZQ175KI mouse models [37]. Here we demonstrate that such MSN preservation and improved spine density are achieved when CYP46A1 is targeted either to neurons or to astrocytes, confirming a non-cell autonomous mechanism for the therapeutic effects of CYP46A1.

Our results showing that specific targeting of astrocytes enhance neuroprotection compared to specific neuronal targeting, suggest that optimal therapeutic effects require CYP46A1 expression in astrocytes and neurons. In a therapeutic perspective, wecompared the outcomes of specific neuronal or astrocytic CYP46A1 targeting to those of a combined strategy using a strong ubiquitous CAG promoter, as previously tested in the R6/2 and the zQ175 mouse models of HD. [36, 37] Our results clearly confirm that the use of the CAG promoter which drives 82% of neuronal expression and only 8% of cellular expression in astrocytes, strongly improves the therapeutic benefit of AAV-CYP46A1 gene therapy in HD as evidenced on MSN preservation, spine density and reduction of mHTT aggregates. Interestingly, the modulation of the cholesterol synthesis was much higher with the use of the CAG promoter compared to the GFA2 or the hSYN vectors. Particularly, a significant increase of 24-OHC and desmosterol, the intermediate compound of the Bloch pathway preferentially used by astrocytes, was observed, consequences on the inflammatory profile were also notably enhanced. The improved therapeutic efficacy observed after AAVrh10-CAG-HA-CYP46A1 injection could be due to the combined expression in neurons and astrocytes, as well as the globally higher levels of CYP46A1 expression, particularly in terms of significant transcriptomic modulation.

## Conclusion

Altogether, our data allow a more comprehensive view of the role of astrocytes in neuroprotection observed after AAV-CYP46A1 delivery and cholesterol pathway activation and highlight the importance of considering the contribution of astrocytes in therapeutic strategies for HD. Synergistic combination of neuroprotective effects with the significant reduction of the toxic mutated HTT protein aggregates by activation of the autophagic clearance represent a strong integrated mechanism of action for AAV-CAG-CYP46A1 gene therapy. 

## Supplementary Information

Supplementary file 1.

## Data Availability

All data generated or analyzed during this study are included in this published article and available from the corresponding author on reasonable request.

## Abbreviations

7-DHC: 7-Dehydrocholesterol
8-DHC: 8-Dehydrocholesterol
24-OHC: 24-Hydroxycholesterol
AD: Alzheimer’s disease
BBB: Blood-brain-barrier
BDNF: Brain-derived neurotrophic factor
BSA: Bovine serum albumin
CAG: Cytosine-adenine-guanosine trinucleotide
CAG: CMV/β-actin hybrid promoter
CYP46A1: Cholesterol 24-hydroxylase
DARPP-32: Medium spiny neurons marker
GC-MS: Gas chromatography-mass spectrometry
GFA2: Glial fibrillary acidic 2 promoter
HD: Huntington’s disease
HMGCR: 3-Hydroxy-3-methylglutaryl-coenzyme A reductase
Hprt1: Hypoxanthine guanine phosphoribosyltransferase 1
hSYN: Human synapsin promoter
IL-6: Interleukin-6
IL-1β: Interleukin-1β
mHTT: Mutant Huntingtin
MPTP: 1-Methyl-4-phenyl-1,2,3,6-tetrahydropyridine
MS: Multiple sclerosis
MSN: Medium spiny neuron
NeuN: Neuronal nuclei marker
NGS: Normal goat serum
PBS: Phosphate-buffered saline
PCA: Principal component analysis
ROI: Region of interest
RT: Room-temperature
SCA3: Spinocerebellar ataxias
SREBP-2: Sterol regulatory element-binding protein-2
TNF: Tumor necrosis factor
TGF: Transforming growth factor
UHDRS: Unified Huntington’s disease rating scale
VCN: Vector copy number
WT: Wild-type
