# Supplementary material for: Astrocyte-neuron combined targeting for CYP46A1 gene therapy in Huntington’s disease
Source: Acta Neuropathol Commun. 2025 Aug 26;13:184. doi: 10.1186/s40478-025-02054-4 (PMC12382279; doi:10.1186/s40478-025-02054-4)
Supplement: Supplementary file 1 [file 40478_2025_2054_MOESM1_ESM.pdf]

SUPPLEMENTAL MATERIALS: SUPPLEMENTAL FIGURES

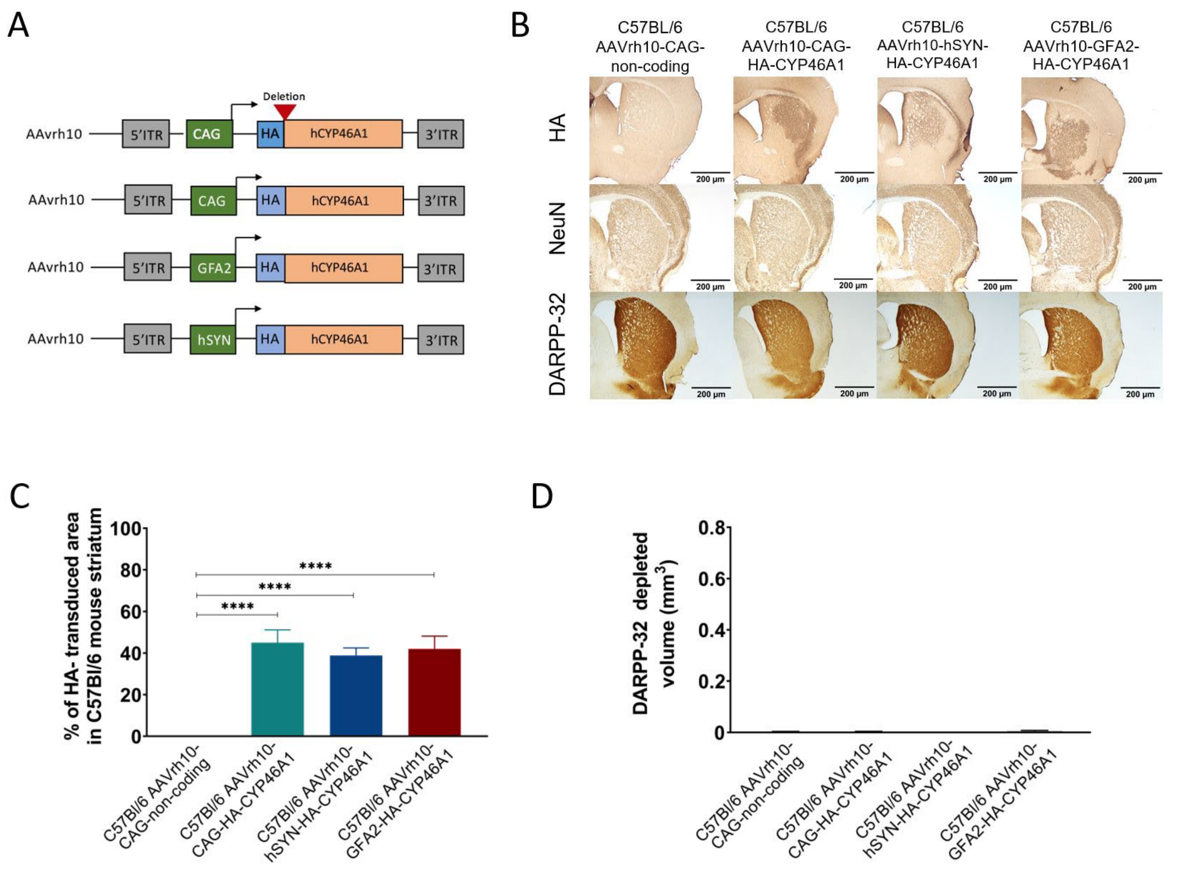

Supplementary Fig. 1: AAVrh10-mediated CYP46A1 delivery in the striatum of C57BL/6J mice, 3 weeks after injection.

(**A**) Schematic representation of AAV constructs enabling the expression of the cassette containing CYP46A1 or not (control sequence that does not express CYP46A1 protein because of a deletion of 145 pb) and the human HA (Hemagglutinin) tag, under the control of the CAG, hSYN or GFA2 promoters.

(**B**) Representative pictures of diaminobenzidine (DAB)-staining using an antibody detecting the HA tag, which labels CYP46A1 protein and DARPP-32 staining and NeuN staining, which labels GABAergic neurons and the nuclei of neurons in coronal brain sections from C57BL/6J mice 3 weeks after injection respectively

(**C**) Quantification of the percentage of the HA immunoreactivity area in the C57BL/6J mouse striatum. No HA immunoreactivity with the non-coding vector was observed. No differences between the different groups injected with the different HA expressing AAV vectors were observed (n=4-5/group). Statistical analysis: One-way ANOVA followed by Dunnett's post-hoc test. Scale bar: 200 µm.

(D) Quantification of the percentage of DARPP-32 depleted volume in C57BL/6J mouse striatum. No depletion of DARPP-32 was observed for all the different groups injected with the different AAV vectors (n=4-5/per group) Statistical analysis: Kruskal-Wallis test followed by Dunn's post-hoc.

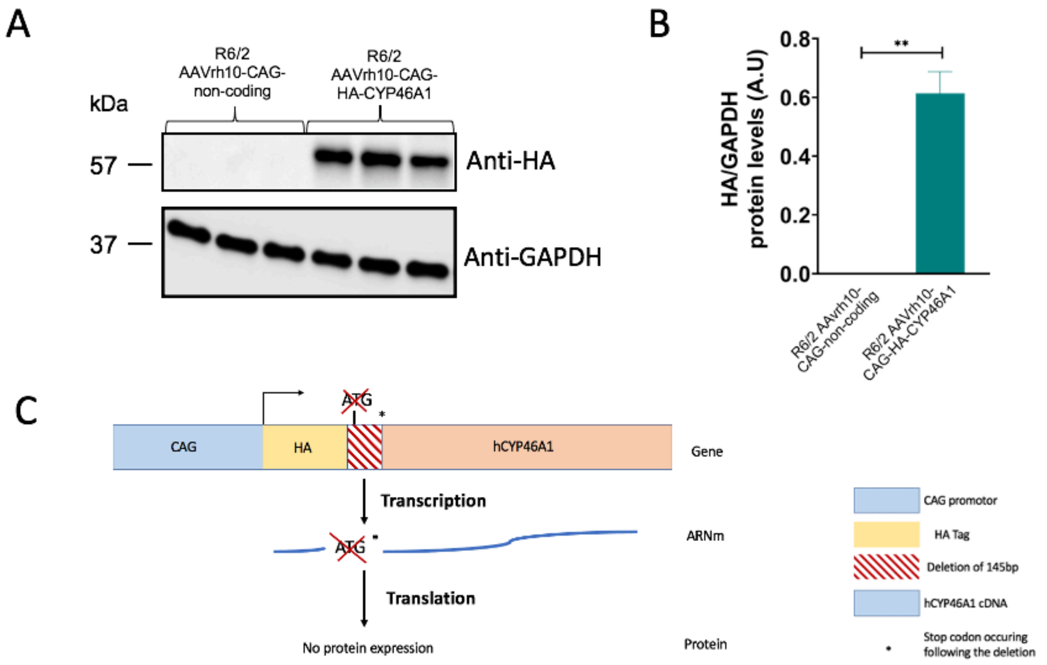

Supplementary Fig 2: AAVrh10-CAG-non-coding does not express transgenic protein in R6/2 mice.

(**A**) Representative cropped-western blot of striatum extracts from 12-week-old R6/2 mice injected with ''AAVrh10-non-coding'' and with AAVrh10-CAG-HA-CYP46A1 constructs. The western-blot membrane was probed with the anti-HA antibody. (**B**) For optical

densitometry quantification, signal intensities were normalized to GAPDH used as a loading control. Data are represented at mean ± S.E.M (n=3/group). Statistical analysis: unpaired t-test: two-tailed $^{**}P$=0.001 relatively to R6/2 ''non-coding'' injected mice. (**C**) Schematic representation of the AAVrh10-CAG-non-coding construct that consists in a frameshift mutation (deletion of 145 pb) in the hCYP46A1 cDNA sequence, producing the mRNA which will be read out of the frame 6 amino acids after the point of the deletion, yielding a nonsense protein.

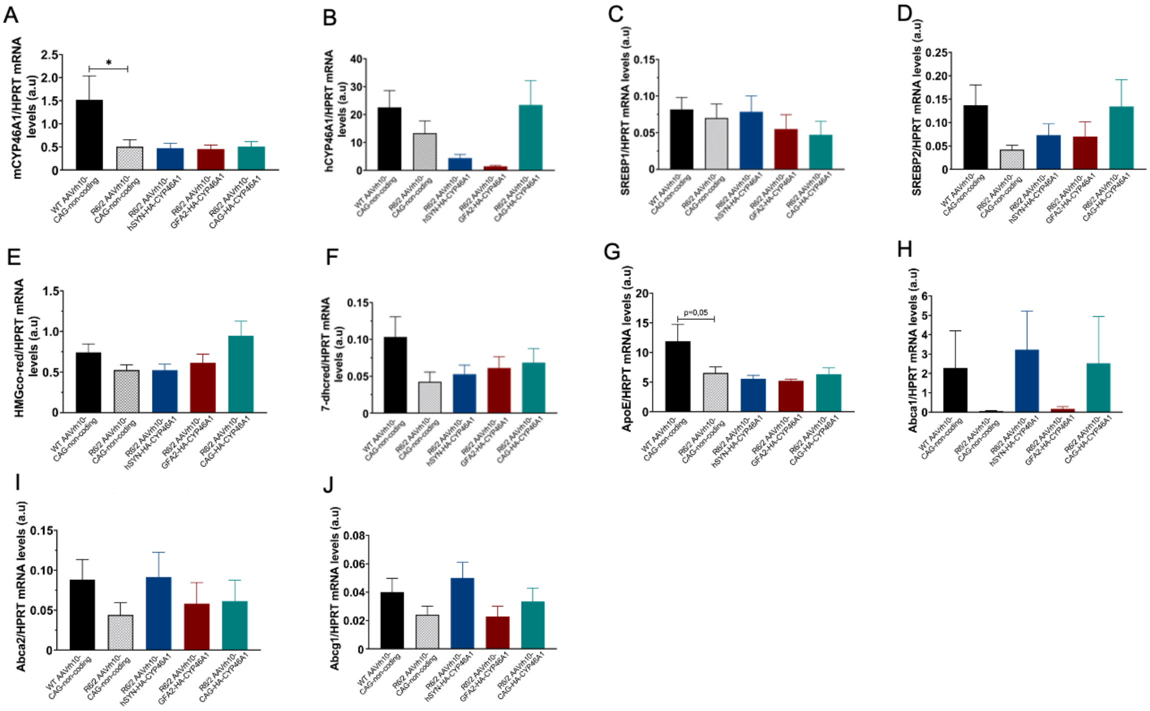

Supplementary Fig 3: Overexpression of CYP46A1 in striatal astrocytes or neurons does not impact mRNA levels of cholesterogenic enzymes in the striatum of R6/2 mice, 8 weeks after injection.

(**A-B**) The mRNA levels of degradation enzymes *mCyp46a1* and h*Cyp46a1* were measured by qPCR from striatal extracts from 12-week-old R6/2 mice and littermates (8 weeks after injection); (**C-D**) transcription factors *Srebp-1* and *Srebp-2*; (**E-F**) synthesis enzymes *HmgCo-A*, *7-dhcred*; (**G-L**) The transcripts for the transporters *ApoE, Abca1, Abca2, Abcg1, Abcg4* were measured by qPCR from striatal extracts from 12-week-old R6/2 mice and littermates. qPCR values were normalized to the *Hprt* housekeeping gene. Data were represented according to the mean ± SEM (n = 4-8 per group). Statistical analysis: One-way ANOVA followed by Dunnett's post-hoc test or Kruskal-Wallis followed by Dunn's post-hoc test (* P <0.05).

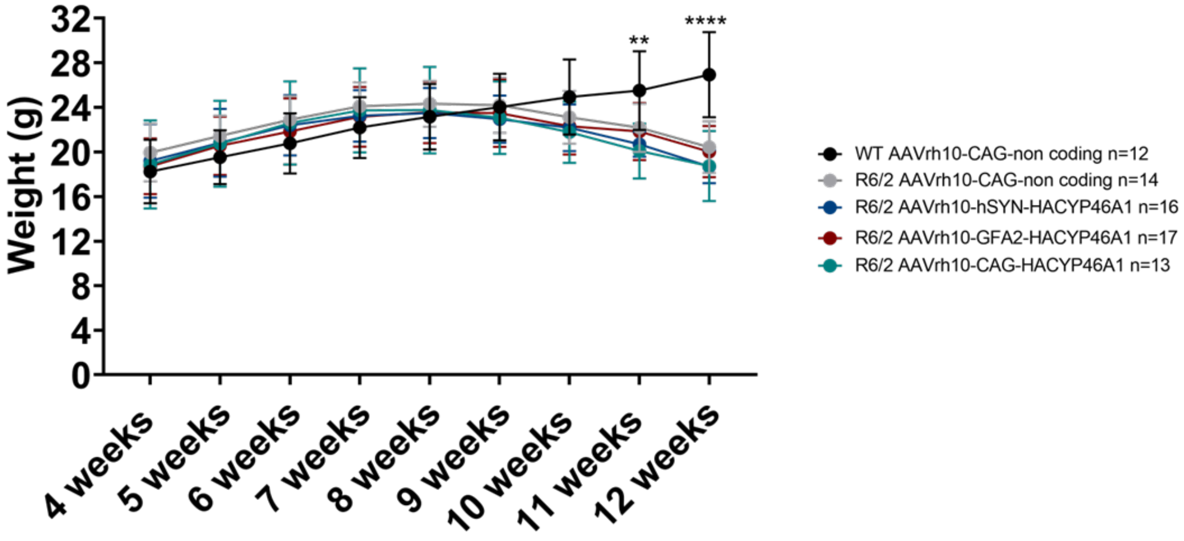

Supplementary Fig. 4: Longitudinal body weight follow-up after HA-CYP46A1 overexpression in the striatum of R6/2 mice using the different constructs.

(A) Body weight was measured from the beginning of the study (week 4) and thereafter once a week until euthanasia (week 12) for animal that underwent behavioral test. Data are presented as mean ± SEM. Statistical analysis: two-way ANOVA followed by Dunnett's post-hoc test (**P <0.01; **** P <0.0001: WT-type non-coding vs R6/2 non-coding).

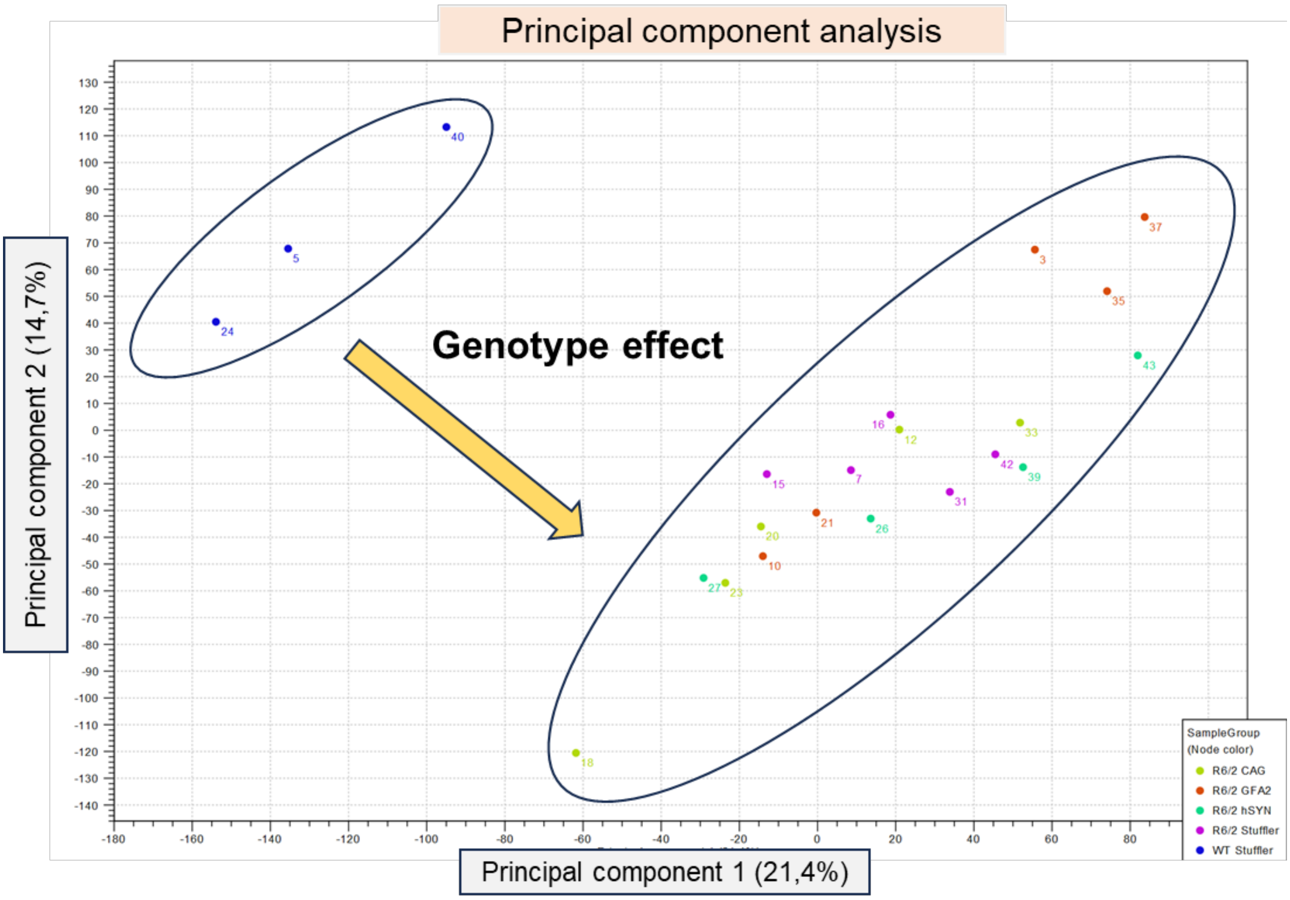

Supplementary Fig 5: Principal component analysis (PCA) score plots of 12 week-old R6/2 and WT littermates samples.

PCA of WT and R6/2 mouse at 12 week-old. Circles highlights the high segregation between WT non-coding mice vs R6/2 groups (=strong genotype effect)

A

Ingenuity Canonical Pathways	-log(p-value)	z-score	Molecules
Gαi Signaling	8,06E00	2,582	ADCY1,ADCY2,ADCY5,ADCY8,ADCY9,ADORA1,ADRA2A,ADRA2B,ADRA2C,AGTR1,APLNR,CHRM2,CHRM4,CNR1,DRD2,DRD3,DRD4,FFAR3,GABBR1,GABBR2,GNA12,GNA14,GNAI2,GNAO1,GNB2,GNB3,GNB5,GNG2,GNG3,GNG4,GNG7,GRM2,GRM3,GRM4,GRM8,HRAS,HRH3,HTR1A,HTR1B,HTR1D,KRAS,LPAR1,MAPK1,MRAS,NRAS,OPRD1,OPRK1,OPRL1,P2RY12,P2RY13,PRKAG2,PRKAR2A,PRKAR2B,PTGER3,RAF1,RAP1GAP,RAP2B,RASD1,RASD2,RGS10,RGS12,RGS14,RGS4,RGS7,RRAS,S1PR1,S1PR3,SHC2,SHC3,SOS1,SOS2
Potassium Channels	7,38E00	5,988	ABCC8,GABBR1,GABBR2,GNB2,GNB3,GNB5,GNG2,GNG3,GNG4,GNG7,HCN2,HCN3,HCN4,KCNA1,KCNA2,KCNA4,KCNA6,KCNAB1,KCNAB2,KCNAB3,KCNB1,KCNC1,KCNC2,KCND2,KCNF1,KCNG4,KCNH3,KCNH4,KCNH8,KCNJ10,KCNJ11,KCNJ12,KCNJ16,KCNJ2,KCNJ4,KCNJ9,KCNK1,KCNK13,KCNK2,KCNK3,KCNK4,KCNK7,KCNK9,KCNMA1,KCNMB2,KCNMB4,KCNN1,KCNQ2,KCNQ3,KCNQ5,KCNS1,KCNS2,KCNS3,KCNV1
Opioid Signaling Pathway	7,26E00	4,237	ADCY1,ADCY2,ADCY5,ADCY8,ADCY9,AKT2,AKT3,CACNA1C,CACNA1E,CACNA1G,CACNA1I,CACNA2D3,CACNA2D4,CACNB1,CACNB2,CACNB3,CACNB4,CACNG1,CACNG2,CACNG3,CACNG4,Calm1 (includes others),CAMK1,CAMK1D,CAMK1G,CAMK2A,CAMK2B,CAMK4,CREB1,CREBBP,EGR4,ELK1,EP300,GNA12,GNA14,GNAI2,GNAO1,GNB2,GNB3,GNB5,GNG2,GNG3,GNG4,GNG7,GRIN1,GRIN2B,GRIN2C,GRK5,GSK3B,HRAS,ITPR1,KCNJ9,KRAS,LCK,LEF1,MAPK1,MAPK12,MAPK4,MAPK6,MRAS,MYC,NOS1,NPBWR1,NRAS,OPRD1,OPRK1,OPRL1,PDE1B,PDK1,PDYN,PENK,PLCB1,PPP3CA,PPP3R1,PRKAG2,PRKAR2A,PRKAR2B,PRKCB,PRKCD,PRKCE,PRKCG,PRKCI,PRKCQ,PRKCZ,PRKD1,RAF1,RAP2B,RASD1,RASD2,RGS10,RGS11,RGS12,RGS13,RGS14,RGS16,RGS19,RGS20,RGS4,RGS6,RGS7,RGS8,RGS9,RPS6KA1,RPS6KA2,RPS6KA3,RPS6KA4,RPS6KA5,RPS6KA6,RPS6KB2,RRAS,RYR1,SOS1,SOS2,SRF,TCF7L1,TCF7L2
cAMP- mediated signaling	6,56E00	5,157	ADCY1,ADCY2,ADCY5,ADCY8,ADCY9,ADORA1,ADORA2A,ADRA2A,ADRA2B,ADRA2C,ADRB1,AGTR1,AKAP3,AKAP5,APEX1,APLNR,Calm1 (includes others),CAMK1,CAMK1D,CAMK1G,CAMK2A,CAMK2B,CAMK4,CHRM1,CHRM2,CHRM3,CHRM4,CNGA2,CNR1,CREB1,CREBBP,CRHR1,DRD1,DRD2,DRD3,DRD4,DRD5,DUSP1,DUSP6,FFAR3,GABBR1,GABBR2,GDE1,GDPD1,GDPD3,GNAI2,GNAO1,GRM2,GRM3,GRM4,GRM8,HRH2,HRH3,HTR1A,HTR1B,HTR1D,HTR4,HTR6,LPAR1,MAPK1,MC5R,OPRD1,OPRK1,OPRL1,P2RY12,P2RY13,PDE10A,PDE1B,PDE4A,PDE5A,PDE6C,PDE6G,PDE7B,PDE8A,PDE9A,PKIA,PKIG,Pld6,PPP3CA,PPP3R1,PRKAR2A,PRKAR2B,PTGER3,RAF1,RAP1GAP,RAPGEF4,RGS10,RGS12,RGS14,RGS2,RGS4,RGS7,RPS6KA1,S1PR1,S1PR3,SMPDL3B,VIPR1,VIPR2
CREB Signaling in Neurons	6,55E00	9,003	ACKR1,ADCY1,ADCY2,ADCY5,ADCY8,ADCY9,ADGRA1,ADGRA2,ADGRA3,ADGRB1,ADGRB2,ADGRD1,ADGRE1,ADGRF4,ADGRG6,ADORA1,ADORA2A,ADRA1A,ADRA1B,ADRA1D,ADRA2A,ADRA2B,ADRA2C,ADRB1,AGTR1,AKT2,AKT3,APLNR,AVPR1B,BMP6,BRS3,CACNA1C,CACNA1E,CACNA1G,CACNA1I,CACNA2D3,CACNA2D4,CACNB1,CACNB2,CACNB3,CACNB4,CACNG1,CACNG2,CACNG3,CACNG4,CALCR,Calm1 (includes others),CAMK2A,CAMK2B,CAMK4,CCKAR,CCKBR,CCR1,CELSR1,CELSR3,CHRM1,CHRM2,CHRM3,CHRM4,CNR1,CREB1,CREBBP,CRHR1,CX3CR1,DRD1,DRD2,DRD3,DRD4,DRD5,EDNRA,EDNRB,EGF,EGFR,ELK1,EP300,FFAR2,FFAR3,FGFR2,FGFR3,FZD10,GABBR1,GABBR2,GLP2R,GNA12,GNA14,GNAI2,GNAO1,GNB2,GNB3,GNB5,GNG2,GNG3,GNG4,GNG7,GPR101,GPR108,GPR12,GPR135,GPR139,GPR146,GPR153,GPR155,GPR158,GPR161,GPR179,GPR26,GPR3,GPR37,GPR37L1,GPR45,GPR50,GPR6,GPR68,GPR75,GPR83,GPR85,GPR88,GPRC5B,GRIA3,GRIA4,GRIK2,GRIK3,GRIN1,GRIN2B,GRIN2C,GRM1,GRM2,GRM3,GRM4,GRM5,GRM8,GTF2B,HCRTR1,HRAS,HRH2,HRH3,HTR1A,HTR1B,HTR1D,HTR2A,HTR2C,HTR4,HTR6,IGF1,IGF2R,ITPR1,KRAS,LPAR1,MAPK1,MAS1,MC5R,MCHR1,MRAS,NGFR,NPBWR1,NPY2R,NRAS,NTRK1,NTRK2,NTRK3,NTSR2,OPRD1,OPRK1,OPRL1,OXGR1,P2RY12,P2RY13,PIK3C2B,PLCB1,PLCB4,PLCD1,PLCD3,PLCH2,PLCL1,PLCZ1,POLR2A,POLR2B,PRKAG2,PRKAR2A,PRKAR2B,PRKCB,PRKCD,PRKCE,PRKCG,PRKCI,PRKCQ,PRKCZ,PRKD1,PTGER3,RAF1,RAP2B,RASD1,RASD2,RPS6KA1,RRAS,RXFP1,S1PR1,S1PR3,S1PR4,SCTR,SHC2,SHC3,SLC52A2,SOS1,SOS2,SSTR2,TACR1,TACR3,TAS1R1,TGFA,TGFBR1,TGFBR2,TRHR,Trhr2,TSHR,VIPR1,VIPR2
Glutaminergic Receptor Signaling Pathway (Enhanced)	6,45E00	6,075	ADCY1,ADCY2,ADCY5,ADCY8,ADCY9,AGK,AKT2,AKT3,BDNF,CACNA1C,CACNA1E,CACNA1G,CACNA1I,CACNA2D3,CACNA2D4,CACNB1,CACNB2,CACNB3,CACNB4,CACNG1,CACNG2,CACNG3,CACNG4,Calm1 (includes others),CAMK2A,CAMK2B,CAMK4,CHUK,CREB1,CREBBP,DGKA,DGKB,DGKD,DGKH,DGKI,DGKK,DGKZ,FMR1,GABBR1,GABBR2,GABRA4,GABRA5,GABRA6,GABRB2,GABRD,GABRQ,GLUL,GNAI2,GNAO1,GOSR1,GPAA1,GRIA3,GRIA4,GRIK2,GRIK3,GRIN1,GRIN2B,GRIN2C,GRM1,GRM2,GRM3,GRM4,GRM5,GRM8,HOMER1,ITPR1,KCNK2,KCNK3,KCNK9,LCAT,MAPK1,MAPK11,MAPK12,MAPK14,NAPA,NAPB,NR3C1,PIK3C2B,PLA2G5,PLA2G7,PLAAT1,PLB1,PLCB1,PLCB4,PLCD1,PLCD3,PLCH2,PLCL1,PLCZ1,PLD3,PLD5,Pld6,PPP3CA,PPP3R1,PRDX6,PRKAG2,PRKAR2A,PRKAR2B,PRKCB,PRKCD,PRKCE,PRKCG,PRKCI,PRKCQ,PRKCZ,PRKD1,RPS6KB2,SCN1B,SCN2B,SCN3B,SCN4B,SCN8A,SCN9A,SHANK1,SHANK3,SLC1A1,SLC1A2,SLC1A6,SLC38A3,SLC38A4,SLC38A5,SLC7A11,SNAP25,STX1A,STX1B,TGFB3,TRPM1,VTI1A,VTI1B
Dopamine-DARPP32 Feedback in cAMP Signaling	6,44E00	3,775	ADCY1,ADCY2,ADCY5,ADCY8,ADCY9,ATP2A1,ATP2A2,CACNA1C,CACNA1E,CACNA2D3,CACNA2D4,CACNB1,CACNB2,CACNB3,CACNB4,CACNG1,CACNG2,CACNG3,CACNG4,Calm1 (includes others),CAMK4,CAMKK1,CAMKK2,CDK5,CREB1,CREBBP,CSNK1D,CSNK1G2,DRD1,DRD2,DRD3,DRD4,DRD5,GNAI2,GRIN1,GRIN2B,GRIN2C,GUCY1A2,ITPR1,KCNJ10,KCNJ11,KCNJ12,KCNJ16,KCNJ2,KCNJ4,KCNJ9,NOS1,PLCB1,PLCB4,PLCD1,PLCD3,PLCH2,PLCL1,PLCZ1,PPP1CA,PPP1CB,PPP1R1B,PPP1R3A,PPP1R3C,PPP1R3D,PPP1R7,PPP2R1B,PPP2R2A,PPP2R2B,PPP2R2C,PPP2R2D,PPP2R3A,PPP2R5A,PPP3CA,PPP3R1,PRKAG2,PRKAR2A,PRKAR2B,PRKCB,PRKCD,PRKCE,PRKCG,PRKCI,PRKCQ,PRKCZ,PRKD1,PRKG2
Role of NFAT in Cardiac Hypertrophy	6,31E00	6,863	ADCY1,ADCY2,ADCY5,ADCY8,ADCY9,AKAP5,AKT2,AKT3,CACNA1C,CACNA1E,CACNA1G,CACNA1I,CACNA2D3,CACNA2D4,CACNB1,CACNB2,CACNB3,CACNB4,CACNG1,CACNG2,CACNG3,CACNG4,Calm1 (includes others),CAMK1,CAMK1D,CAMK1G,CAMK2A,CAMK2B,CAMK4,CTF1,EP300,GNAI2,GNB2,GNB3,GNB5,GNG2,GNG3,GNG4,GNG7,GSK3B,HDAC1,HDAC2,HDAC5,HDAC6,HDAC9,HRAS,IGF1,ITPR1,KRAS,LIF,MAPK1,MAPK10,MAPK11,MAPK12,MAPK14,MEF2C,MRAS,NRAS,PIK3C2B,PLCB1,PLCB4,PLCD1,PLCD3,PLCH2,PLCL1,PLCZ1,PPP3CA,PPP3R1,PRKAG2,PRKAR2A,PRKAR2B,PRKCB,PRKCD,PRKCE,PRKCG,PRKCI,PRKCQ,PRKCZ,PRKD1,RAF1,RAP2B,RASD1,RASD2,RCAN2,RCAN3,RRAS,SHC2,SHC3,SLC8A2,SLC8A3,SOS1,SOS2,TGFB3,TGFBR1,TGFBR2
Calcium Signaling	6,18E00	5,515	ACTA1,AKAP5,ATP2A1,ATP2A2,ATP2B1,ATP2B2,CACNA1C,CACNA1E,CACNA1G,CACNA1I,CACNA2D3,CACNA2D4,CACNB1,CACNB2,CACNB3,CACNB4,CACNG1,CACNG2,CACNG3,CACNG4,Calm1 (includes others),CAMK1,CAMK1D,CAMK1G,CAMK2A,CAMK2B,CAMK4,CAMKK1,CAMKK2,CHRNA1,CHRNA10,CHRNA3,CHRNA4,CHRNA5,CHRNA7,CHRNB3,CREB1,CREBBP,EP300,GRIA3,GRIA4,GRIN1,GRIN2B,GRIN2C,HDAC1,HDAC2,HDAC5,HDAC6,HDAC9,ITPR1,MAPK1,MEF2C,MYH1,MYH10,MYH13,MYH14,MYH2,MYH3,MYH4,MYH7B,MYH8,MYL1,MYL4,MYL6B,MYO18A,MYO1A,MYO5C,NFATC3,PNCK,PPP3CA,PPP3R1,PRKAG2,PRKAR2A,PRKAR2B,RAP2B,RCAN2,RCAN3,RYR1,SLC8A2,SLC8A3,TNNC1,TNNI3,TNNT1,TNNT2,Tpm1,Tpm2,Trpc2,TRPC3,TRPC4,TRPC6,TRPC7,TRPV6
Synaptic Long Term Potentiation	5,57E00	4,106	ADCY1,ADCY8,CACNA1C,Calm1 (includes others),CAMK2A,CAMK2B,CAMK4,CREB1,CREBBP,EP300,GNA14,GRIA3,GRIA4,GRIN1,GRIN2B,GRIN2C,GRM1,GRM2,GRM3,GRM4,GRM5,GRM8,HRAS,ITPR1,KRAS,MAPK1,MRAS,NRAS,PLCB1,PLCB4,PLCD1,PLCD3,PLCH2,PLCL1,PLCZ1,PPP1CA,PPP1CB,PPP1R1A,PPP1R3A,PPP1R3C,PPP1R3D,PPP1R7,PPP3CA,PPP3R1,PRKAG2,PRKAR2A,PRKAR2B,PRKCB,PRKCD,PRKCE,PRKCG,PRKCI,PRKCQ,PRKCZ,PRKD1,RAF1,RAP2B,RASD1,RASD2,RPS6KA1,RRAS

B

Molecules Name
1700020N18Rik
GPR35
IFI16
LY9
LYPD1
NMS
OPN3
PDCD1
SATB2
SLC11A1
SP110
WNT10A

C

	IPA CANONICAL PATHWAYS Z-score	R6/2 non-coding vs WT non-coding	WT non-coding vs R6/2 non-coding	R6/2 non-coding vs R6/2 hSYN-CYP	R6/2 non-coding vs R6/2 GFA2-CYP	R6/2 non-coding vs R6/2 CAG-CYP
	Huntington's Disease Signaling	-3,286	3,286		-0,447	0,577
**Cholesterol biosynthesis pathways**	Cholesterol biosynthesis	-3,207	3,207			3,638
	Cholesterol Biosynthesis I	-2,449	2,449			3
	Cholesterol Biosynthesis II (via 24,25-dihydrolanosterol)	-2,449	2,449			3
	Cholesterol Biosynthesis III (via Desmosterol)	-2,449	2,449			3
	Activation of gene expression by SREBF (SREBP)	-1,147	1,147			2,84
	DHCR24 Signaling Pathway	-4,621	4,621	-0,447	0	3,441
	Mevalonate Pathway I	-1,633	1,633			2,449
	Superpathway of Cholesterol Biosynthesis	-2,887	2,887			4
	Superpathway of Geranylgeranyldiphosphate Biosynthesis I (via Mevalonate)	-1,633	1,633			2,646
	PPAR Signaling	0,928	-0,928		-0,333	-3,051
**Synaptogenesis & Synaptic plastic pathways**	Activation of NMDA receptors and postsynaptic events	-3,8	3,8		0	2,236
	BMP signaling pathway	-2,117	2,117			2,121
	Calcium Signaling	-5,515	5,515		0,816	1
	CREB Signaling in Neurons	-9,003	9,003	-1,414	-0,354	4,036
	cAMP-mediated signaling	-5,157	5,157	0	-0,577	2,132
	Dopamine-DARPP32 Feedback in cAMP Signaling	-3,775	3,775		-1,134	2,333
	G alpha (q) signalling events	-4,808	4,808	0,378	0	2,985
	GABA receptor activation	-3,272	3,272			2,714
	GP6 Signaling Pathway	-3,667	3,667		-1,155	4,025
	Gαi Signaling	-2,582	2,582		0,378	1,698
	Gαq Signaling	-3,81	3,81	-1	0,632	2,333
	HIF1α Signaling	-4,276	4,276		-0,447	2,449
	Myelination Signaling Pathway	-2,55	2,55	-0,905	-1,789	2,197
	Neuregulin Signaling	-2,92	2,92		0	2,236
	NGF Signaling	-2,111	2,111		-1,342	2,121
	P2Y Purigenic Receptor Signaling Pathway	-4,422	4,422		-1,134	3,464
	Phospholipase C Signaling	-2,75	2,75		-0,905	3,578
	Potassium Channels	-5,988	5,988	2	-1	4,472
	RHO GTPases Activate NADPH Oxidases	-2,828	2,828			2,449
	RHOGDI Signaling	2,598	-2,598		1	-2,111
	SNARE Signaling Pathway	-5,077	5,077		1	2,496
	Signaling by Rho Family GTPases	-3,906	3,906		-1,387	2,982
	Synaptic Long Term Depression	-5,969	5,969		0	3
	Synaptic Long Term Potentiation	-4,106	4,106		-0,447	2,309
	Synaptogenesis Signaling Pathway	-7,121	7,121	0	-1,387	3,9
**Inflammation pathways**	Neuroinflammation Signaling Pathway	-2,734	2,734	-3,606	-1,789	4,323
	IL-2 Signaling	-1,606	1,606			2,828
	IL-3 Signaling	-2,646	2,646		-1,342	2,309
	IL-4 Signaling	-2,887	2,887		-0,535	4,802
	IL-6 Signaling	-1,976	1,976		-0,577	3,638
	IL-8 Signaling	-4,16	4,16	-2,236	-0,632	3,638
	IL-10 Signaling	0	0	0	-1,886	-2,268
	IL-15 Signaling	-3,128	3,128			2,714
	Interleukin-10 signaling	-1,342	1,342	-2,449	0,447	3,742
	Interleukin-2 family signaling	-0,577	0,577			3
	S100 Family Signaling Pathway	-9,328	9,328	-1,633	-0,469	5,674
	STAT3 Pathway	-2,92	2,92		1	1,414
	TGF-β Signaling	-0,73	0,73			2,309
	Toll-like Receptor Cascades					2,828
	Toll-like Receptor Signaling	-0,302	0,302			2,646
	SAPK/JNK Signaling	-2,121	2,121		0,447	3,051
	CDC42 Signaling	-1,279	1,279		-0,707	3,162
	CDK5 Signaling	-1,677	1,677		-1	2,887
	Chemokine Signaling	-3,402	3,402			2,828
	NF-κB Signaling	-3,317	3,317	-2	0,905	3,922

D

		HD EFFECT		CYP46A1 EEFECT (AAVrh10-CAG)		ROLE
		FC	p-value	FC	p-value	
Cholesterol metabolism	ACAT2	Down 1,5	8,59E-05	UPx1,3	1,27E-03	Conversion of Acetyl-CoA into Aceto-Acetyl-CoA
	HMGCS1	Down x 2	6,68E-10	UP x3	2,06E-11	Conversion of Acetoacetyl-CoA into HMGC-CoA
	HMGCR	Down x1,3	1,04E-02	UP x1,4	1,26E-08	Conversion of Acetyl-coA into Mevalonate
	MVK	No changes	5,26E-01	UP x1,3	1,68E-02	Conversion of Mevalonate into Mevalonate-5-P
	MVD	Down x1,5	8,39E-03	UP1,4	1,53E-02	Conversion of Mevalonate 5-P into isopentenyl-5-pyrophsphate
	FDPS	No changes	3,55E-01	UPx1,5	2,28E-05	Conversion of isopentenyl-PP into Geranyl PP and Farnesyl-PP
	IDI1	Down x1,9	1,14E-03	UP x2,4	7,81E-05	Conversion of Isopentenyl-PP into Dimethyl-allyl-PP
	FDFT1	Down 1,5	7,74E-09	UPx1,9	3,93E-09	Conversion of farnensyl-PP into squalene
	SQLE	Downx1,6	5,28E-04	UP x2,4	6,63E-14	Conversion of Squalene to 2,3-Epoxysqualene
	LSS	No changes	3,16E-01	UP x1,3	5,29E-04	Conversion 2,3-Epoxysqualene to lanosterol
	CYP51	Down 1,9	7,74E-09	UPx1,7	7,00E-08	Conversation of lanosterol into 4,4-demethyl-5 alpha cholesta-8-14-24-trien-3-beta-ol
	MSMO1	Down x 2	3,05E-13	UP x2	1,63E-16	conversion of 4,4-dimethylzymosterol into Zymosterol
	SC5D	No changes	1,10E-01	UP x1,3	8,98E-03	conversion of 4,4-dimethylzymosterol into Zymosterol
	DHCR 7	No changes	6,22E-02	**No changes**	**5,61E-02**	Conversion of 7-dehydrocholesterol into cholesterol
	DHCR24	Down x 2	1,94E-05	UP x2	1,26E-08	Conversion of desmosterol into cholesterol
	CYP46A1	No Changes	1,14E-01	UPx1,5	2,65E-02	Conversion of cholesterol in 24-OHC
	SREBPF1	Upx 1,3	3,76E-02	Down x 1,3	1,26E-02	Transcription factor regulating the expression of genes involved in global lipid synthesis and growth
	SREBPF2	Down x,8	5,65E-05	UP x1,9	1,75E-07	Transcription factor regulating the expression of genes involved in cholesterol regulation
Huntington's disease signalling	NEUROD1	No changes	5,02E-01	Up x4,5	2,70E-03	Neuron survival and growth factor
	BDNF	Down x5	2,19E-03	UP x4	3,48E-03	Neuron survival and growth factor
	HDAC 1	UP x2	1,11E-06	Down x1,3	2,37E-02	Histone deacetylase 1
	HAP1	UP x2	3,27E-06	Down x1,4	1,26E-03	Huntington-associated protein
Immune response	CXCR3	No changes	3,35E-01	UP x4,5	1,73E-02	Chemokine receptor
	TgFb1	No changes	3,35E-01	UP x4,5	1,73E-02	Growth factor
	TGFBR1	Down x 1,4	8,53E-04	Down x1,3	2,48E-03	Growth factor receptor
	TGFBR2	Down x1,5	4,48E-03	Down 1,5	3,21E-03	Growth factor receptor
UBIQUITIN PROTEASOME	Ube2L6	No Changes	4,42E-01	UP x1,6	4,59E-02	Ubiquitin conjugating enzyme
	Psmb9	No Changes	2,01E-01	UP x2,7	2,12E-03	20S proteasome subunit
	Psmb8	No Changes	6,24E-01	UP x2	3,92E-03	20S proteasome subunit
	Psmb5	No Changes	2,68E-01	UPx1,4	8,65E-03	20S proteasome subunit
Cell survival	Parp3	DOWN 1,5	2,46E-02	UPx1,6	4,22E-03	DNA repair
	Atf5	No Changes	4,68E-01	UP x1,3	2,38E-02	Transcription factor associated to cell survival
Autophagy	Acp1	No Changes	8,75E-01	UPx4	1,25E-03	Endosomal recycling
	Acp5	Down x3,2	1,92E-02	UPx4	1,25E-03	Lysosome function
	Lamp5	No changes	1,79E-01	Down x1,3	1,06E-02	Lysosome function

Supplementary Fig 6 : Close-up on striatal overexpression of CYP46A1 under action of CAG, hSYN and GFA2 promoters on cholesterol biosynthesis, inflammation cascades, synaptogenesis, and LTP/LTD pathways, 8 weeks after injection.

(**A**) Detailed description of the molecules associated with top 10 IPA canonical pathways WT-CAG-non-coding vs R6/2 CAG-non-coding. (**B**) Name of the 12 common DEGs overlap between all coding vectors and R6/2-CAG-non-conding vs R6/2 AAVrh10-GFA2-HA-

CYP46A1 and R6/2-CAG-non-coding vs R6/2 AAVrh10-hSYN-HA-CYP46A. (**C**) Pathways significantly altered (*P*<0.05) at 3 months: R6/2 CAG-non-coding compared to WT-CAG-non-coding; and R6/2 AAVrh10-CAG-HA-CYP46A1, AAVrh10-GFA2-HA-CYP46A1, or R6/2 AAVrh10-hSYN-HA-CYP46A1 compared to R6/2 CAG-non-coding. Cholesterol pathways are highlighted in green. IPA z-score indicates if the pathway is predicted to be inhibited (z-score <-2), activated (z-score >2) or activation or inhibition cannot be predicted (grey). (D) Table of target genes related to cholesterol metabolism, inflammation, autophagy, ubiquitin, proteasome, growth factor which illustrate the main transcriptional modification after CYP46A1 expression.

The column "HD effect" shows the down or up regulations observed in the R6/2 non-coding mice compared to WT-littermates' mice. The column "CYP46A1 effect" shows the down or up regulations observed in the R6/2 mice injected with CYP46A1 compared R6/2 non-coding mice.

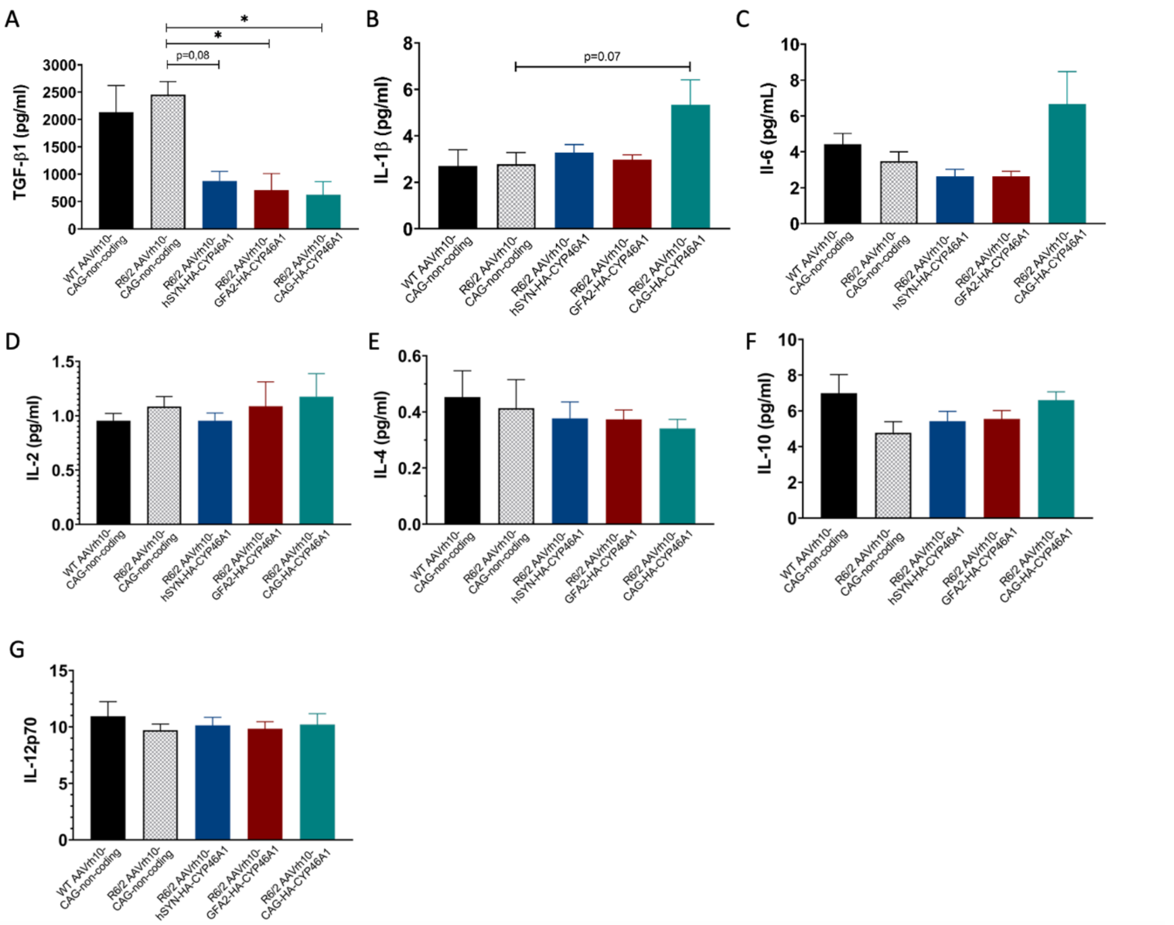

Supplementary Figure 7: CYP46A1 overexpression in R6/2 mouse striatum modulate the levels of specific cytokines and interleukins, 8 weeks after injection.

(**A-G**) ELISA quantification (MSD immunoassay) of cytokines and interleukins levels in the striatum of 12-week-old WT-littermates and control R6/2 mice ‘’non-coding’’, R6/2 injected with CYP46A1 coding vectors mediated by CAG, hSYN and GFA2 promoters. (**A**) AAVrh10-CAG-HA-CYP46A1 and AAVrh10-GFA2-HA-CYP46A1 lead to a statistically significant decrease of TGF-β1 compared to control group [* P <0.05 (n=4-11/group)], while a trend was observed for AAVrh10-hSYN-HA-CYP46A1[P=0.08]. A non-statistical significant trend for increase was observed in R6/2 mice injected with AAVrh10-CAG-CYP46A1 for IL-6 (n=4-11/group) **(B)** and Il-1β (n=4-9/group) [P=0.07] **(C),** compared to control mice No differences were observed among groups for (**D**) IL-2 (n=6-12/group), (**E**) IL-4 (n=3-9/group), (**F**) IL-10 (n=6-12/group), **(G)** IL-12p70 (n=6-12/group Data are represented as mean ± SEM. Statistical

analysis: One-way ANOVA followed by Dunnett's post-hoc test or Kruskal-Wallis followed by Dunn's post-hoc test.

Unmodified full-length Western Blots

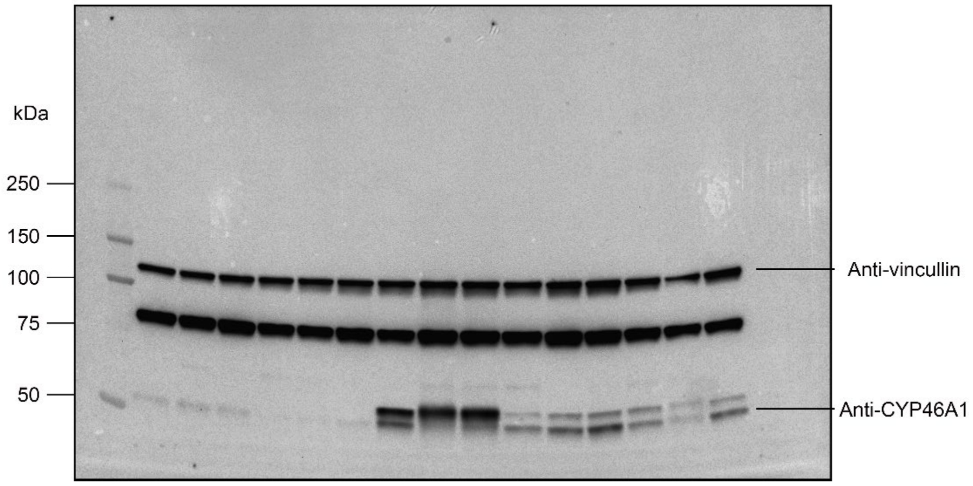

Related to Figure 2:

Unmodified full-length western blot of total CYP46A1 levels in striatal extracts from 12-week-old R6/2 mice and injected with the different constructs and age-matched WT-littermates (8 weeks after injection). For optical densitometry quantification signal intensities were normalized to vinculin protein, used as loading control.

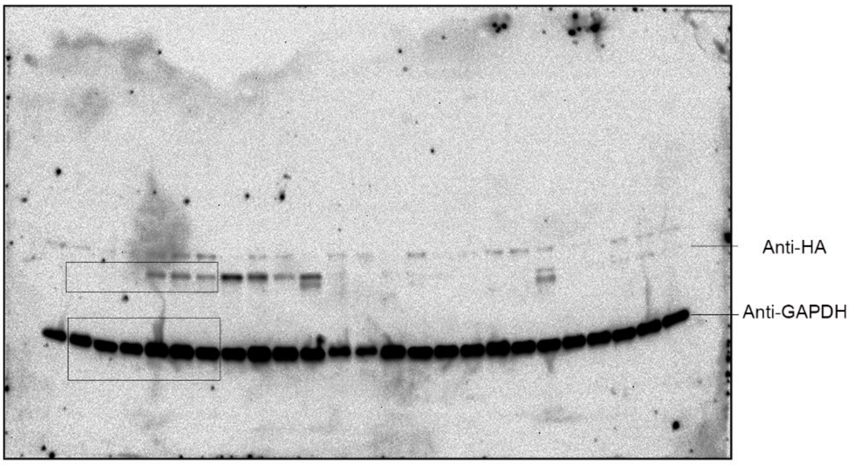

Related to Supplementary Fig 2: AAVrh10-CAG-non-coding does not express transgenic protein in R6/2 mice.

Unmodified full-length western blot of striatum extracts from 12-week-old R6/2 mice injected with ''AAVrh10-non-coding'' and with AAVrh10-CAG-CYP46A1 constructs. The western-blot membrane was probed with the anti-HA antibody. For optical densitometry quantification, signal intensities were normalized to GAPDH used as a loading control. The black boxes indicates which part of the blot was cropped.

SUPPLEMENTAL MATERIALS: MATERIAL AND METHODS

Image acquisition for neuropathological assessment in C56BL/6J mice

Images of immunostained sections were captured with a brightfield Leica DM 6000B microscope. Photographs for comparison were taken under identical conditions of image acquisition, and all adjustments of brightness and contrast were applied uniformly for each staining. Using *ImageJ* version 1.53c (NIH, Bethesda, USA), HA immunoreactivity was quantified [(Σstained area / Σtotal striatal area) x100]. Besides, measurement of potential neuronal loss in frozen brain sections was based on NeuN and DARPP-32 immunostained slices. For each brain slice, NeuN+ or DARPP32+ area was compared to the total area of the striatum. A percentage of depleted area per mouse was calculated [(Σtransduced area / Σtotal striatal area) x100]. To avoid potential technical artefacts laser power, numeric gain and magnification are kept constant between animals. In order to the respective staining, images were first converted to 8-bit grey scale. The area of stained striatum (or depleted area) and the area of total striatum were measured for each section and then added per mouse. All these analyses were carried out on 7 to 9 slices around the injection site for each mouse.

Cholesterol and oxysterol measurements

Mouse striatal tissue samples were weighed and homogenized with a TissueLyser II apparatus (Qiagen) in a 500 ml solution containing butylated hydroxytoluene (BHT, 50 mg/ml) and

EDTA (0.5 M). At this point, a mix of internal standards was added [epicoprostanol, 2H7-7-lathosterol, 2H6-desmosterol, 2H6-lanosterol and 2H7-24(R/S)-hydroxycholesterol] (Avanti Polar Lipids). Alkaline hydrolysis was performed under Ar using 0.35 M ethanolic KOH for 2h at room temperature (RT). After neutralization of the solution with phosphoric acid, sterols were extracted in chloroform. The lower phase was collected, dried under a stream of nitrogen and the residue was dissolved in toluene. Oxysterols were then separated from the cholesterol and its precursors on a 100 mg Isolute silica cartridge (Biotage); cholesterol was eluted in 0.5% propan-2-ol in hexane followed by oxysterols in 30% propan-2-ol in hexane. The sterol and oxysterol fractions were independently silylated with Regisil_ + 10% TMCS [bis(trimethylsilyl) trifluoroacetamide + 10% trimethylchlorosilane] (Regis technologies) as described previously.[125] The trimethylsilylether derivatives of sterols and oxysterols were separated by gas chromatography (Hewlett-Packard 6890 series) in a medium polarity capillary column RTX-65 (65% diphenyl 35% dimethyl polysiloxane, length 30 m, diameter 0.32 mm, film thickness 0.25 mm; Restesk). The mass spectrometer (Agilent 5975 inert XL) in series with the gas chromatography was set up for detection of positive ions. Ions were produced in the electron impact mode at 70 eV. They were identified by the fragmentogram in the scanning mode and quantified by selective monitoring of the specific ions after normalization and calibration with the appropriate internal and external standards [Epicoprostanol m/z 370, 2H7-7-Lathosterol m/z 465, 2H6-Desmosterol m/z 358, 2H6-Lanosterol m/z 504, 2H7-24(R/S)-Hydroxycholesterol m/z 413, Cholesterol m/z 329, 7-Lathosterol m/z 458, 7-Dehydrocholesterol m/z 325, 8-Dehydrocholesterol m/z 325, Desmosterol m/z 351, Lanosterol m/z 393 and 24(R/S)-Hydroxycholesterol m/z 413]. If needed, coeluted peaks were integrated using a dedicated deconvolution macro (amdistyty.mac) provided by Agilent.

ELISA assay for interleukins and cytokines detection

IL-1-ß, IL-2, IL4, IL-6, IL-10, IL-12p70 were measured using the U-plex Biomarker Group 1 (ms) Assays, MSD (Mesoscale Discovery, USA). TGF- β1 was measured using U-plex Mouse TGF-β1 Assay MSD (Mesoscale Discovery, USA). ELISA assays were performed following supplier instructions. The data were analyzed using MSD Workbench software. The software fits the standard curves using a 4-parameter logistic (PL) fit with 1/y2 weighting. The 4-PL equation is: y=signal; x=concentration; b2=estimated response at infinite concentration; b1=estimated response at zero concentration, b3=mid-range concentration, b4=slope factor. Values under the LLOD (lower limit of detection were not included in the analysis). LLOD corresponds to the lowest analyte concentration that is significantly above background. In MSD Workbench software, LLOD is determined as the analyte concentration equivalent to the signal that is 2.5x SD above the backfit signals of the blank.
